# Suzuki–Miyaura cross-coupling optimization enabled by automated feedback†
†Electronic supplementary information (ESI) available: Details regarding system operation and optimization method, optimization data, and spectroscopic data. See DOI: 10.1039/c6re00153j



**DOI:** 10.1039/c6re00153j

**Published:** 2016-10-18

**Authors:** Brandon J. Reizman, Yi-Ming Wang, Stephen L. Buchwald, Klavs F. Jensen

**Affiliations:** a Department of Chemical Engineering , Novartis-MIT Center for Continuous Manufacturing , Massachusetts Institute of Technology , 77 Massachusetts Avenue , Cambridge , MA 02139 , USA . Email: kfjensen@mit.edu; b Department of Chemistry , Novartis-MIT Center for Continuous Manufacturing , Massachusetts Institute of Technology , 77 Massachusetts Avenue , Cambridge , MA 02139 , USA . Email: sbuchwal@mit.edu

## Abstract

An automated, droplet-flow microfluidic system explores and optimizes Pd-catalyzed Suzuki–Miyaura cross-coupling reactions. A smart optimal DoE-based algorithm is implemented to increase the turnover number and yield of the catalytic system considering both discrete variables—palladacycle and ligand—and continuous variables—temperature, time, and loading—simultaneously. The use of feedback allows for experiments to be run with catalysts and under conditions more likely to produce an optimum; consequently complex reaction optimizations are completed within 96 experiments. Response surfaces predicting reaction performance near the optima are generated and validated. From the screening results, shared attributes of successful precatalysts are identified, leading to improved understanding of the influence of ligand selection upon transmetalation and oxidative addition in the reaction mechanism. Dialkylbiarylphosphine, trialkylphosphine, and bidentate ligands are assessed.

## Abstract 2

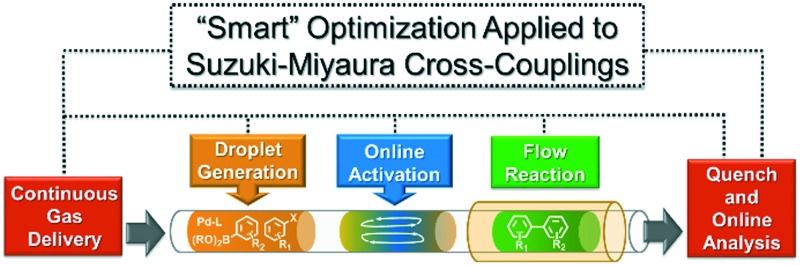
An automated, droplet-flow microfluidic system explores and optimizes Pd-catalyzed Suzuki–Miyaura cross-coupling reactions.

## Introduction

Heralding the era of the “robo-chemist”,^1^ automated chemical development continues to gain prominence in academia and industry, ushering in novel ways to synthesize libraries, optimize reaction conditions, and evaluate kinetics.^2,3^ Such growth in popularity, however, has been met with equally growing skepticism^4^ that the job of the chemist could ever truly be replaced with a robot. Among the reasons given are that:

• Automated technologies imported from biology lack the generality to handle the wide diversity of reagents and process conditions managed by the process chemists.

• Although automated experiments can be run in parallel, analysis becomes a time consuming and expensive bottleneck.

• Though a robot can run many more experiments than a chemist, the chemist's intuition on what experiment is best to run may be more valuable than any number of uninformative screening experiments.

Drawing especially from this last point, recent research has steered not necessarily in the direction of replacing the chemist with automation but instead in using automation to help guide the decision-making process of the chemist, helping to minimize the number of reactions necessary to achieve a satisfactory result.^5^ To this end, there has been an emergence of automated feedback systems for reaction development which use flow chemistry^6^ together with real-time analytical data to optimize reactions in lieu of undirected screening.^7^ The use of flow allows for accurate control of reaction conditions, mixing, and heat transfer^8^ and facilitates access to more hazardous and/or extreme conditions than those that could be achieved in batch.^9^ Optimization algorithms controlling reaction conditions and interpreting online data can direct the system toward higher yields^10^ and improved understanding of reaction kinetics.^11^

Though advantageous in conserving time and starting material and ensuring that experiments are run where maximal information can be obtained, automated flow feedback systems have historically fallen short as valuable tools to a process chemistry lab. Although one reason for this is the implicit flow chemistry knowledge needed to reconfigure components such as syringe pumps, valves and fittings, and reactors to answer different questions for different types of chemistry, a main limitation of adoption of flow optimization is that the only variables that can be studied with such an approach efficiently are continuous variables such as time, temperature, and amount. Discrete variables such as catalyst, ligand, or solvent, which may be most critical to reaction performance and mechanistic insights, are ignored in feedback optimization.

Discrete variable selection and optimization can be achieved using droplet microfluidics,^12^ although generality is again not often addressed in making droplet generation a tool with widespread utility in solving organic chemistry problems. Advantageously, the use of droplets allows for microfluidic reaction volumes to be created using interchangeable reactants, catalysts, and solvents. These model larger scale batch reactors by way of efficient mixing and heat transfer,^13^ while dispersion and the risk of contamination across experiments remains low.

Prevalent as proofs-of-concept, droplet screening systems have in the past faced numerous material compatibility challenges that limit their versatility in chemical development. For instance, simplified droplet-flow systems are often implemented with reaction droplets suspended in an inert perfluorinated carrier phase,^14,15^ which becomes miscible with a majority of commonly used organic solvents at elevated temperatures^16^ and can lead to contamination from experiment to experiment with droplet budding and breaking.^17^ Polydimethylsiloxane, the favored medium for droplet flow devices,^12,18^ swells upon exposure to conventional reagents and solvents. Though UV-vis,^19^ fluorescence,^15,20^ and IR^21^ are all popular analytical methods for demonstrating the speed of droplet screening, these do not achieve the resolution of chromatographic methods when it comes to distinguishing key products and intermediates.

Recently, we demonstrated a microfluidic flow screening system that assessed a diverse array of organic solvents for amine alkylation over a range of temperatures, flow rates, and species concentrations.^22^ The system utilized nitrogen as an inert carrier and a Teflon tube microreactor for broad reagent and temperature versatility. Rather than a comprehensive screen, a smart algorithm was employed that optimized continuous variables simultaneously with the elimination of candidate solvents; consequently only 93 automated experiments were required to determine the optimum among 10 solvents and three reaction variables.

**Scheme 1:**
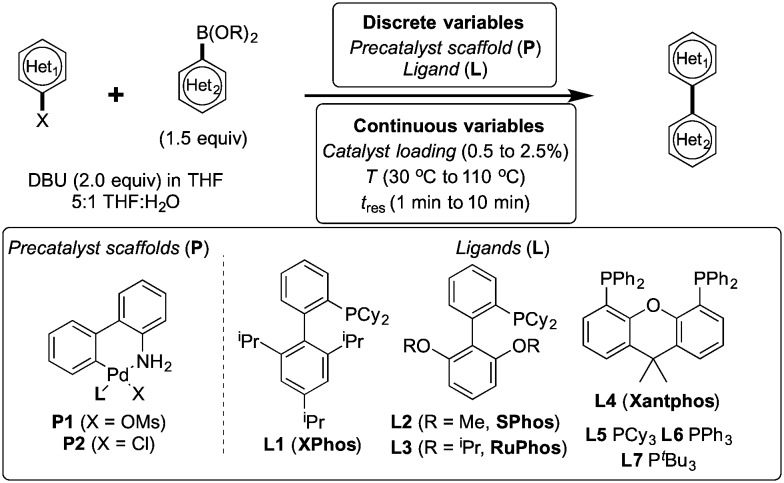
Optimization scheme for Suzuki–Miyaura cross-couplings in the presence of 1,8-diazabicyclo[5.4.0]undec-7-ene (DBU) and THF/water.

To showcase the complexity of chemical reaction system handled by such a platform and feedback optimization approach, we explored optimization of several case studies of Suzuki–Miyaura cross-coupling reactions involving heterocyclic substrates (Scheme 1).^23^ Suzuki–Miyaura couplings are among the most utilized reactions in organic chemistry, particularly in the pharmaceutical industry.^24^ Despite advances in ligand development and mechanistic understanding of the Suzuki–Miyaura coupling, choosing the right catalyst/ligand system for a given pair of coupling partners remains a nontrivial task, inspiring research in both high-throughput experimental^3^ and computational^25^ screening of catalyst-ligand systems. Though generations of catalyst precursors and ligands have been developed and iteratively improved to afford wider substrate scope, higher yields, and better turnover numbers,^26,27^ identification of the optimal ligand in conjunction with conditions such as temperature, reaction time, and catalyst loading nevertheless remains largely empirical in nature.

Herein, we examine not only optimization of these systems but rationalization of the results in terms of substrate and precatalyst tendencies, which can be probed more directly with the same automated experimentation platform. With the information gained, a more rational selection of reaction parameters, including the nature of the precatalysts and ligand, should be forthcoming. The case studies presented consider the family of palladacycle-ligand precatalyst systems shown in Scheme 1.^27,28^ We reasoned that the rapid, quantitative activation of these precatalysts upon exposure to base would make them suitable for our flow study. Moreover, these precatalysts have seen wide use in both academia and in industrial settings.^29^

## Methods

**Fig. 1:**
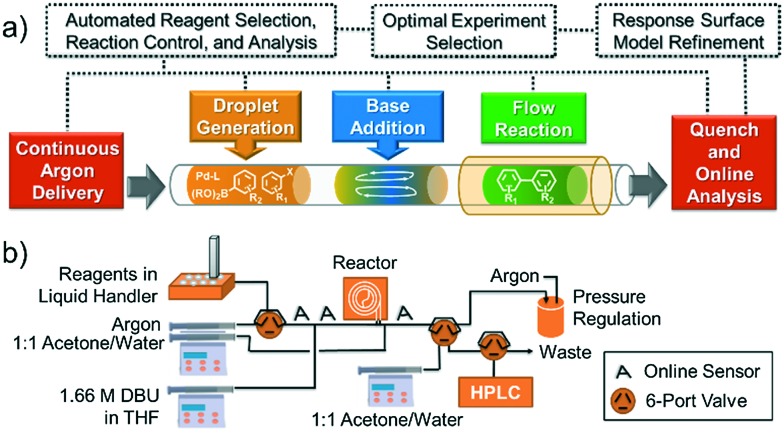
(a) Concept and (b) flow diagram for automated Suzuki–Miyaura cross-coupling optimization. See ESI† Fig. S1 for a complete system process and instrumentation diagram.

Concept and flow diagrams for system operation are provided in Fig. 1, and comprehensive operation and optimization protocols are provided in the ESI.† As a general procedure, precatalysts were synthesized and purified according to the procedure published by Bruno *et al.* ^27^ Samples of precatalysts, excess ligands, aryl halide and internal standard, and boronic acid or boronic pinacol ester were prepared independently in THF and stored under argon in an automated liquid hander (Gilson GX-271) along with neat solutions of THF and water. The remaining steps of the method were performed automatically in a feedback loop for each optimization case study. Following instructions given by the computer algorithm, the automated liquid handler prepared a droplet by sampling and mixing a combination of stock solutions to achieve the desired reagent concentration and catalyst loading. The droplet was injected into the continuous flow system through a 14 μL sample loop and pushed by 6.9 bar argon at a flow rate controlled *via* syringe pump (Harvard Apparatus PhD 2000 with 8 mL Harvard stainless steel syringe). To initiate the reaction, 3.5 μL of 1.66 M DBU in THF were injected into the droplet through a T-junction, and the droplet was delivered to a heated Teflon tube reactor maintained under 6.9 bar argon. At the outlet of the reactor, the reaction was quenched with a 1 : 1 solution of water and acetone, with 1 μL of the diluted droplet sampled in a dual sample loop HPLC injection system (Gilson Valvemate II followed by Agilent G1158A). This sample was filtered and split further in a 1 : 7 ratio between two reversed-phase HPLC columns (Agilent Zorbax SB-C18 2.1 × 50 mm, 4.6 μm particle diameter and 1.8 μm particle diameter), with the smaller sample used for quantitation by UV (Agilent G1365C multi-wavelength detector) and mass verification by MS (Agilent 6120 quadrupole). Between reaction droplets, 14 μL droplets of water, acetone, and THF were introduced into the flow system to wash away any residual reaction material, and the automated droplet preparation, reaction, and analysis procedure was repeated.

To control and optimize the system, we developed software in LabVIEW (National Instruments, ver. 8.6) and MATLAB (The MathWorks, Inc., ver. R2011a) that iteratively formulated response surface models and proposed experiments given candidate discrete variables (precatalysts, ligands), continuous variable ranges (temperature, reaction time, catalyst loading), and online HPLC data. Variables were randomized and all discrete variables were treated as yes/no decisions (*i.e.* shared catalyst attributes and postulated relationships with continuous variables did not factor into the algorithm's calculations). A randomized fractional factorial design of experiments was first proposed by the system, followed by a second fractional factorial design in a targeted region of the continuous variable space. Following these initial experiments, the optimization program constructed response surfaces for each precatalyst comprising aggregate continuous variable linear, interaction, and quadratic response factors, as well as independent temperature and pre-exponential offsets for each discrete variable set. This construct allowed the algorithm to build general knowledge of the interplay of continuous variables in early experimentation while classifying superiority of some precatalysts over others. Ensuing experiments were then selected to challenge candidate optima predicted by the response surface models using the G-optimality criterion,^30^ ensuring that models were refined to minimize uncertainty in the predicted best result for each precatalyst. Minimization of this uncertainty then allowed candidate catalysts to be statistically eliminated from the optimization. As more information was collected and fewer candidate precatalysts were under consideration, experiments became concentrated with the precatalysts and at the conditions most likely to produce the overall optimum. Optimization routines were limited to 96 experiments.

The general catalytic system optimization procedure considered a fixed ratio of 5 : 1 THF to water and temperatures and residence times ranging from 30–110 °C and from 1–10 min, respectively. Catalyst loading ranged from 0.5–2.5 mol%. When studying the effect of excess ligand, temperature was varied in the same 30–110 °C range while the excess ligand equivalents ranged from 0.0–2.0. Aiming to capture the tradeoff between yield and catalyst consumption, the optimization sought to maximize turnover number (TON) (defined herein as the moles product generated divided by moles catalyst used) with a constraint that the reaction yield be greater than 90% of the maximum yield. Such a constrained optimization approach we hypothesized could be easily correlated to the constraint of other reagents or byproducts in future studies. By optimizing for TON with the constraint on yield, an optimum was found that was independent of product calibration—hence no prior product isolation was required.

## Results

### Optimal catalytic system selection

**Fig. 2:**
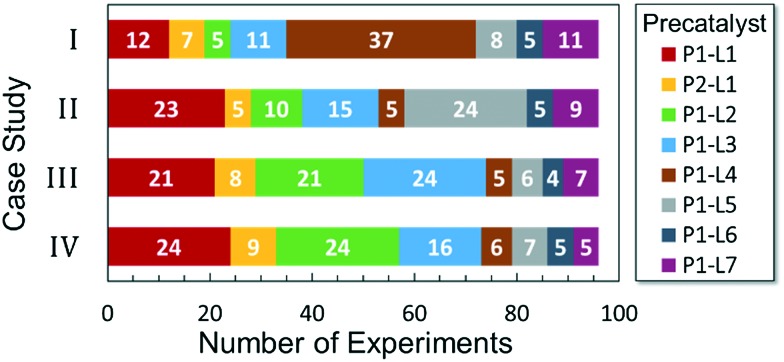
Precatalyst selection frequency by case study.

Optimized results are presented in Table 1. Fig. 2 illustrates how the preference for experimentation with different precatalysts changed given the substrate selection.

**Table 1:** Optimal yield and TON found by automated optimization of Suzuki–Miyaura case studies

	Ar–X	Ar–B(OR)_2_	Optimal conditions	Yield	Max TON
I	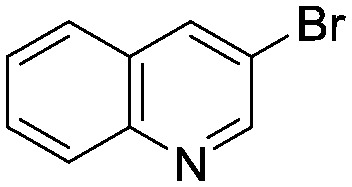 (**1**)	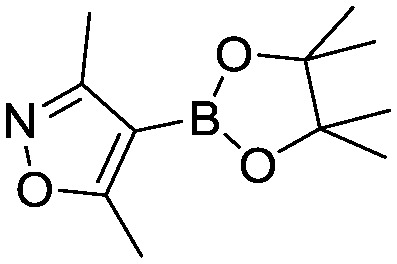 (**2**)	10 min 110 °C 1.2% **P1-L4**	82% (**3**)	69
II	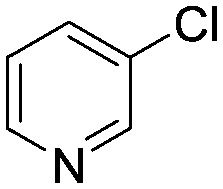 (**4**)	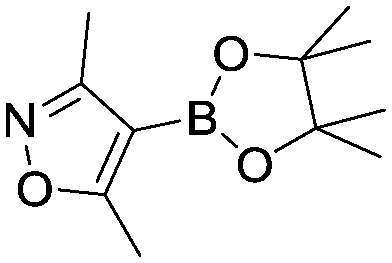 (**2**)	10 min 110 °C 2.1% **P1-L5**	35% (**5**)	17
III	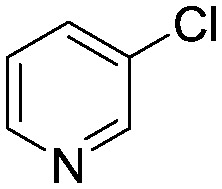 (**4**)	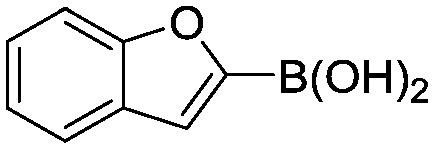 (**6**)	3.9 min 110 °C 1.2% **P1-L1**	88% (**7**)	75
IV	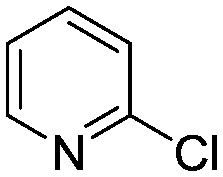 (**8**)	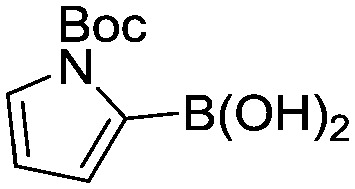 (**9**)	4.7 min 97 °C 1.0% **P1-L1**	90% (**10**)	89

**Fig. 3:**
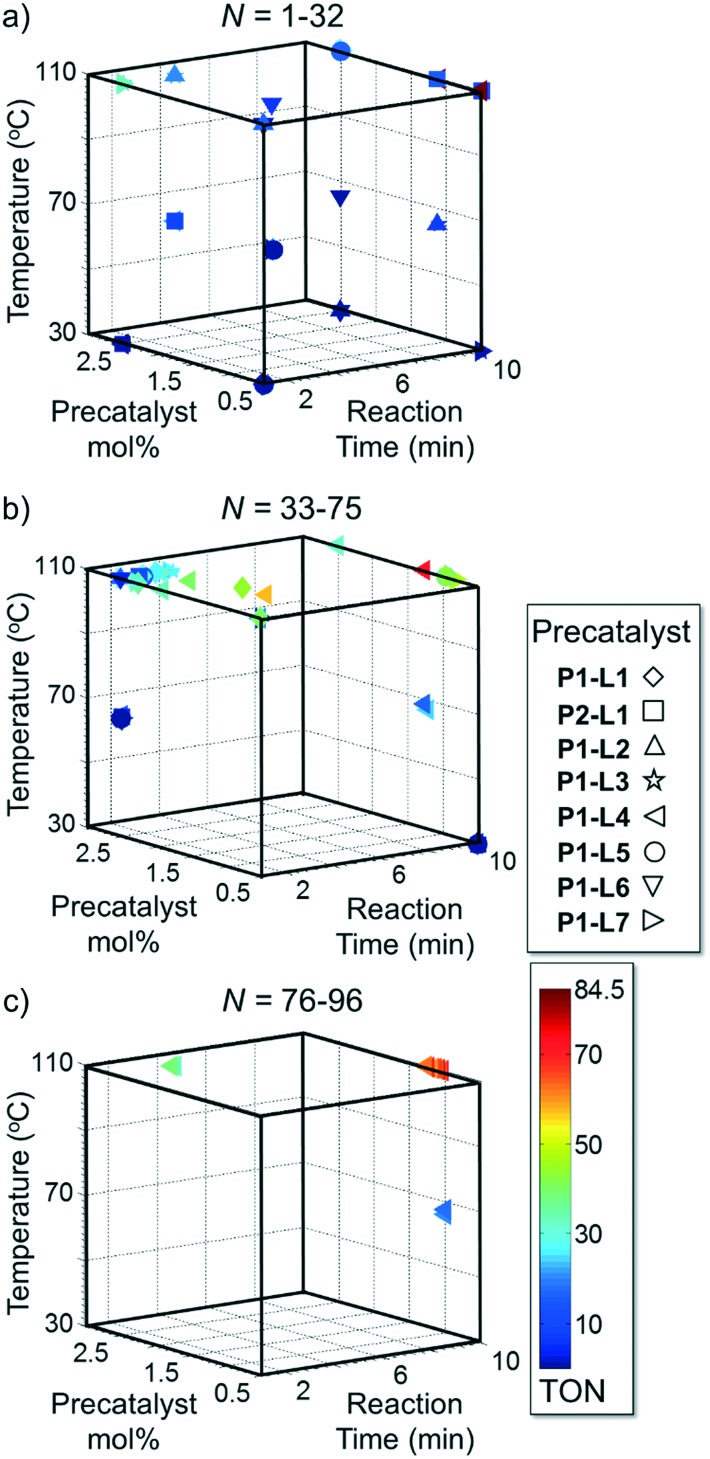
Optimization trajectories followed by the automated system for case I. a) DoE initialization (32 experiments); b) quadratic response surface refinement and discrete variable elimination (39 experiments); c) further response surface refinement with **P1-L4** and convergence (21 experiments).

Case I, the coupling of 3-bromoquinoline (**1**) with 3,5-dimethylisoxazole-4-boronic acid pinacol ester (**2**), was chosen as a model reaction as it provided an example of a challenging Suzuki–Miyaura coupling reaction involving the coupling of two heteroaryl substrates. Experience has taught us that the use of “complex” substrate combinations is usually more predictive of generality than when simple ones (*e.g.*, phenyl boronic acid and 4-bromo- or chloroanisole).^31^ The rapid convergence of the method can be seen from the results in Fig. 3, which shows the progression of the optimization in three stages (though in practice the algorithm moved continuously from one stage to the next). The system initiated by searching the extremes of the continuous variable experimental space (Fig. 3a) before moving to interior points and progressively eliminating candidate precatalysts at conditions that were expected to generate **3** in improved yield and TON (Fig. 3b). These experiments led to iterative refinement of the response surface model until only **P1**-xantphos (**P1-L4**) remained under consideration after 75 experiments. The final experiments conducted by the automated system were directed at tuning the catalyst loading until an optimal TON and an acceptable yield were achieved (Fig. 3c).

**Fig. 4:**
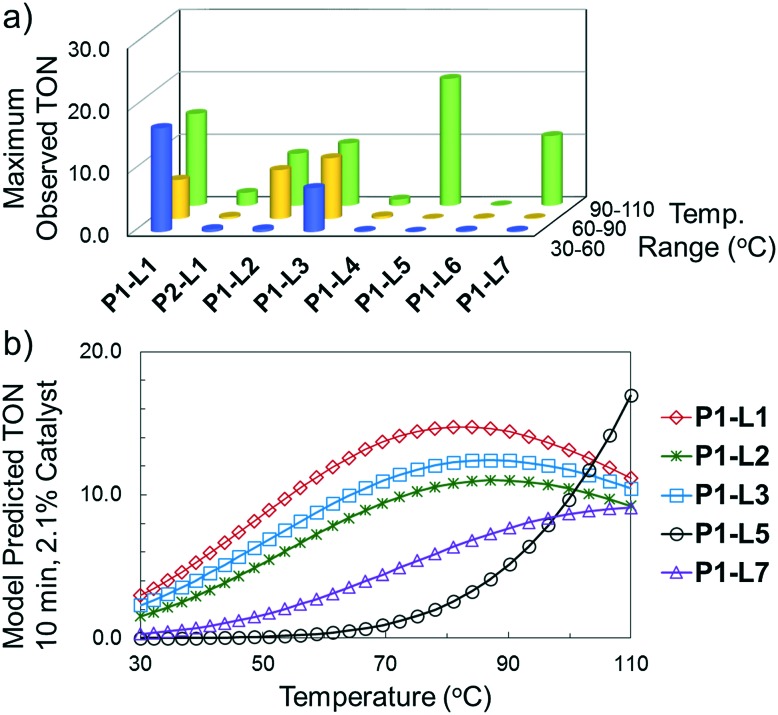
For case II, (a) maximum observed TON during optimization for each precatalyst as a function of temperature range (blue—30–60 °C, yellow—60–90 °C, green—90–110 °C) and (b) final response surface models predicting TON as a function of temperature for five best-performing precatalysts.

The reaction of 3-chloropyridine (**4**) with **2** (case II) was considerably slower than that with **1** and gave poor yields when **P1-L4** was selected as the precatalyst. Among the candidate precatalysts, the system instead identified the precatalyst based on PCy_3_ (**P1-L5**) as optimal, generating 35% yield of **5** in 10 min at 110 °C and 2.1% Pd loading. Though use of the dialkylbiarylphosphine ligands XPhos (**L1**), SPhos (**L2**), and RuPhos (**L3**) with the **P1** precatalyst scaffold gave coupling product **5** in modest yield at lower temperatures, use of **P1-L5** at 110 °C provided the best results (Fig. 4).

**Fig. 5:**
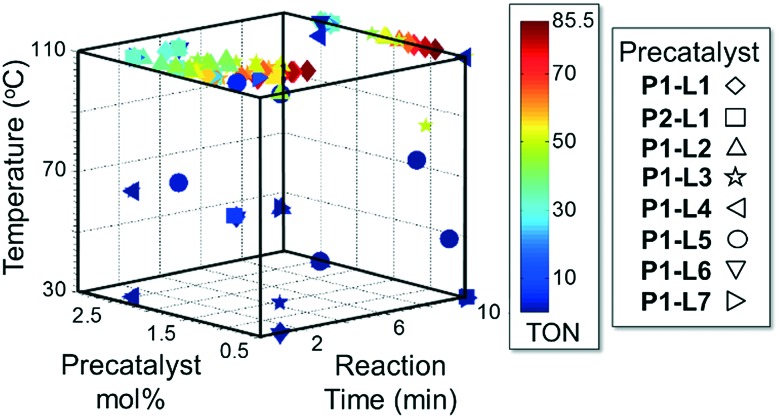
Optimization trajectory for case III.

Abbreviated reaction times were found to be optimal in the cross-couplings of **4** with benzofuran-2-boronic acid (**6**) (case III) and of 2-chloropyridine (**8**) with 1-Boc-2-pyrroleboronic acid (**9**) (case IV), presumably due to the enhanced tendency of **6** and **9** to undergo competitive protodeboronation. In these instances it became especially important to have a fast catalyst to “outrun” this destructive side reaction. In case III, the system rapidly diagnosed that no improvement in TON could be achieved at greater than 3.9 min, and thus experiments were concentrated in the range of reaction times between 1 and 4 min (Fig. 5). The dialkylbiarylphosphine ligands **L1**, **L2**, and **L3** all performed well in the production of **7**, with the system eventually converging to 1.2% **P1-L1** as optimal with respect to TON. Due to the thermal Boc-deprotection of adduct **10** in case IV,^32^ fine-tuning of both reaction time and temperature was necessary to achieve high yields of desired product in the coupling of **8** and **9**. Following an extensive search of the experimental space, the automated system in this case identified 1.0% **P1-L1** as optimal, furnishing **10** in 90% yield in 4.6 min at 97 °C.

**Fig. 6:**
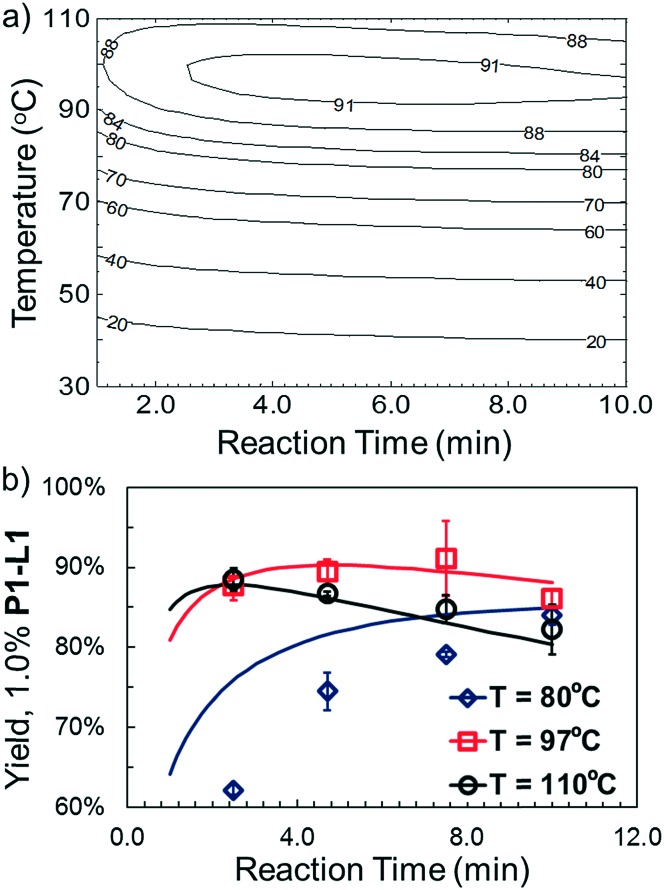
For case IV with 1.0% **P1-L1**: (a) response surface for catalytic TON extracted from optimization; (b) overlay of automated screening experiments (markers) upon response surface predictions (solid line).

The droplet flow reaction approach was further applied in validation of the optimal synthetic conditions for **10**. For 1.0% **P1-L1**, the best-fit response surface generated from “smart” screening (Fig. 6a) showed a region of optimality between 3 and 7 min at less than 100 °C. Fig. 6b overlays this model prediction on a more conventional 3 by 4 grid of droplet flow screening experiments conducted at 80 °C, 97 °C, and 110 °C using 1.0% **P1-L1**. The response surface predictions agreed closely with the screening results near the optimum of 4.7 min and 97 °C and captured the reduced TON at long residence times and high temperature. A comparable yield was observed at 7.5 min and 97 °C; however, these conditions resulted in a greater variance between runs. At 80 °C, the approximated response surface significantly overestimated the yield of the coupling reaction. This inaccuracy was an expected limitation of conducting a higher density of experiments at the optimum; as in the case of all regression-based algorithms, extrapolation to less-explored regions of the experimental space introduced greater uncertainty into the model prediction.^33^ Nevertheless, the rapid and unambiguous identification of a non-trivial reaction time and temperature optimum (*i.e.*, in the interior of the range explored) provides an excellent demonstration of the utility and efficiency of this system. Moreover, an experiment performed in batch validated the applicability of the identified conditions on a scale (1 mmol) useful to the bench chemist (see the ESI† for details).

### Optimization of ligand equivalents

**Scheme 2:**
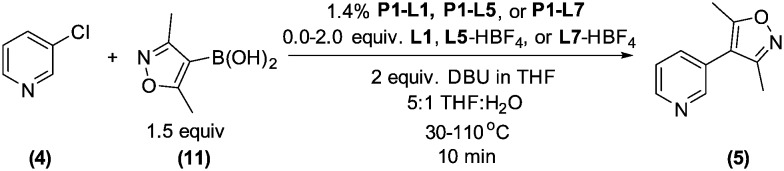
Optimization of temperature, ligand selection, and added ligand equivalents in the synthesis of **5**.

We queried whether the addition of excess ligand would accelerate reactions involving the aryl chloride **4**. For example, the presence of an increased amount of ligand should lead to greater catalyst stability. However, in many cases, this consideration must be balanced by the tendency of excess ligand to decrease the accessibility of the active catalyst through formation of the less reactive L_2_Pd(0) and L_2_Pd(ii) intermediates rather than the more reactive monoligated complexes.^34^ To study this possibility, we employed the same automated system and characterized the reaction of **4** with 3,5-dimethylisoxazoleboronic acid (**11**) (Scheme 2). As a simplification, we considered only the precatalyst **P1** and the ligands **L1**, **L5**, and **L7**, and examined the effect of manipulating temperature and excess equivalents of ligand (from 0.0 to 2.0) at 10 min reaction time and 1.4% precatalyst-ligand loading.

**Fig. 7:**
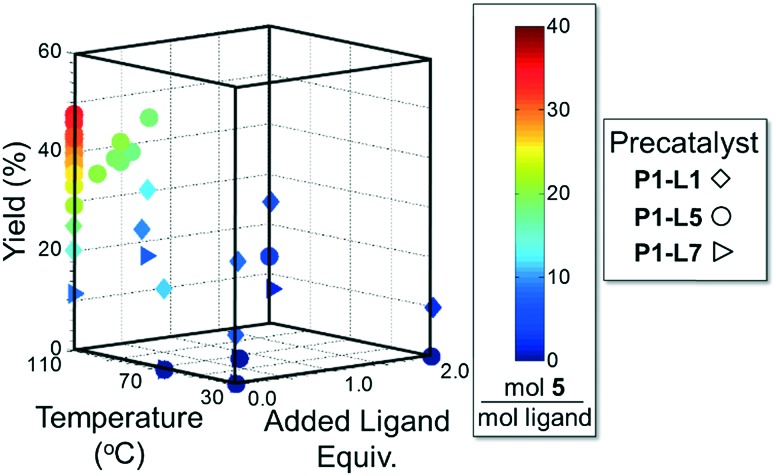
Optimization trajectory for Scheme 2 on the basis of moles **5** per total moles ligand.

As in the cross-coupling of **4** with the boronic acid pinacol ester **2**, the optimization algorithm rapidly identified **P1-L5** as the best catalytic system at 110 °C. Shown in Fig. 7, the reaction yield was somewhat improved in the range of 0.2–0.8 excess ligand equivalents but decreased significantly with the use of 2 excess ligand equivalents for all precatalysts. On a per-ligand basis, it was found to be non-optimal to introduce excess **L5**-HBF_4_ to the **P1-L5** system. For both **L1** and **L7** the automated system indicated the use of only 0.3 excess ligand equivalents as optimal both on a per ligand basis and for the overall reaction yield (see the ESI† for details). Requiring 41 runs, this set of experiments demonstrated the efficiency of our method in answering a non-trivial question regarding the optimal amount of ligand to use and the differences to expect with the substitution of one precatalyst for another.

## Discussion

In the context of the Suzuki–Miyaura cross-coupling mechanism,^35^ we reasoned for case I that a more rapid oxidative addition step for electron-rich dialkylbiarylphosphine or trialkylphosphine ligands did not confer an advantage over **L4** in terms of rate or yield. This result is consistent with the ability of the aryl bromide **1** to readily undergo oxidative addition with a variety of ligands. On the other hand, the poor performance of **L4** in case II may be attributed to the reluctance of **L4**–Pd(0) complexes to undergo oxidative addition to aryl chlorides,^36^ while electron-rich ligands—as expected—afforded significantly higher yields of the desired product.

**Fig. 8:**
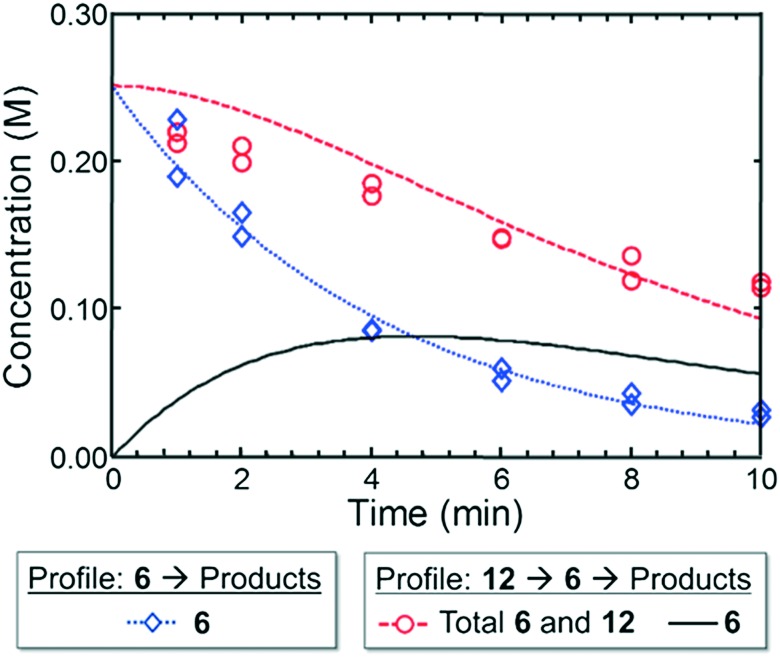
Experimental (markers) and model-fit (lines) kinetic profiles for **6** and **12** at 110 °C. Blue curve and diamonds – evolution of benzofuran-2-boronic acid (**6**) starting with 0.25 M **6**. Red curve and circles – evolution of the combined benzofuran-2-boronic acid pinacol ester (**12**) and **6** starting with 0.25 M **12**. Black curve – model-predicted evolution of intermediate **6** starting with 0.25 M **12** (see the ESI† for model assumptions).

Moving from the boronic pinacol ester **2** to the boronic acids **6** and **9**, a clear transition was observed in the preference of dialkylbiarylphosphine ligands over other ligands considered in the study. An examination of the rate of background protodeboronation of boronic acid **6** in flow indicated a half-life of less than 4 min at 110 °C (Fig. 8), hence the optimality of a shorter reaction time in case III. We attributed the preferences for dialkylbiarylphosphine ligands in this case to faster oxidative addition and rapid transmetalation to L_1_Pd(Ar)Cl intermediates when these ligands were employed, which allowed the desired coupling process to outcompete decomposition pathways.

To provide support for this notion, we conducted flow experiments for case III using **12**, the pinacol ester of boronic acid **6**, which was anticipated to be less susceptible to protodeboronation. Using the automated system, we first measured the combined amount of **6** and **12** remaining as a function of time at 110 °C, under the conditions previously employed to examine the protodeboronation of **6** (Fig. 8, red curve).‡
‡The pinacol boronate **12** was found to undergo complete hydrolysis to the boronic acid **6** during HPLC analysis. Thus the combined amount of **12** and **6** was detected and quantified as **6**. Combining these results with the measurements of the rate of protodeboronation of **6** (Fig. 8, blue curve), we proposed a pseudo-first order kinetic model that allowed for estimation of the availability of boronic acid **6** over time (see the ESI†). This model assumed that only the free boronic acid underwent protodeboronation and was supported by the agreement in the fit of the model to the experimental data. Shown in Fig. 8 (black curve), the use of pinacol boronate **12** effected a controlled release of **6**, resulting in a nearly constant concentration of **6** between 2 and 10 min. This we hypothesized would result in a more substantial increase in yield of the coupling product for a slower catalyst compared to a faster one.

**Scheme 3:**
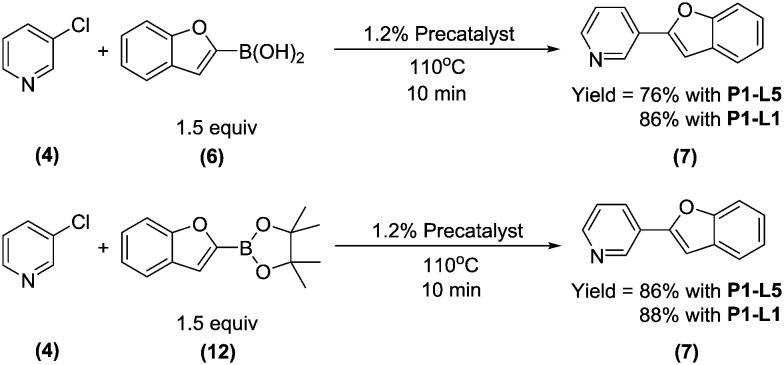
Effect of 2-benzofuranboron reagent and precatalyst upon synthesis of **7**.

To test this hypothesis, we compared the yields of **7** for the coupling of **4** with boronic acid **6** and boronate ester **12** using either **P1-L1** or **P1-L5** as the precatalyst (Scheme 3). To maximize conversion to **7**, all four reactions were performed with the longest studied residence time (10 min). As anticipated, there was an improvement in the yield of **7** upon switching from **6** to **12** as the starting material (76% to 86%) when **P1-L5** was used as the precatalyst. In contrast, the yield was essentially unchanged when **P1-L1** was used. We rationalized that slow turnover of the **L5**-based catalyst together with the rapid protodeboronation of **6** led to the unproductive consumption of the boronic acid and a reduced yield. The use of pinacol boronate **12** in place of **6** resulted in a good matching of the relative rates of catalyst turnover and the release of **6**, thus allowing the boron reagent to be efficiently coupled. In contrast, the catalyst based on **L1** could outpace the rapid protodeboronation of **6**, permitting the efficient use of the free boronic acid in spite of its short lifetime.

**Fig. 9:**
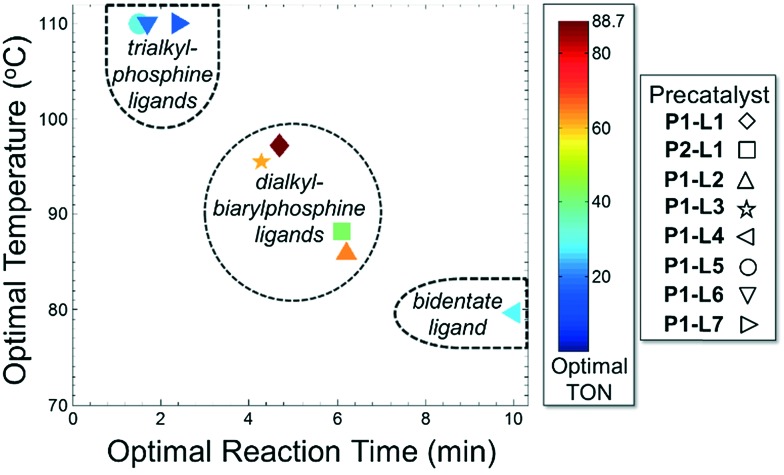
Optimal conditions for case IV.

For case IV, comparison of ligand class with the optimal reaction conditions revealed a distinct segregation in the optimality of the dialkylbiarylphosphine ligands (Fig. 9). Whereas catalyst precursors bound to simple trialkyl- or triarylphosphine ligands (**L5–L7**) were found to be optimal at the maximum temperature and short reaction times, the four dialkylbiarylphosphine precatalysts were found to be optimal in the range of 85–97 °C and at longer reaction times of 4–6 min. That it was possible to use a reduced temperature and shorter reaction time when dialkylbiarylphosphine ligands were employed proved advantageous in the case of a thermally sensitive reactant and product, unlike for the more thermally stable starting materials and products encountered in case I or II.

## Conclusions

Our studies herein have shown the importance of dialkylbiarylphosphine ligands in promoting high catalytic turnover in the presence of aryl chloride substrates and unstable boronic acids and products. With boronic acid pinacol esters, where the rate of hydrolysis to the boronic acid can be limiting, the advantage of using the dialkylbiarylphosphine ligand may, in some cases, be lessened. The same observation can apply in the case of aryl bromides, for which oxidative addition is facile for several other classes of ligands. Heuristically, the data of these case studies show that the choice of the **P1**-XPhos (**P1-L1**) precatalyst is favorable in most cases, but optimality cannot be guaranteed without a comprehensive search of all palladium precursor-ligand combinations. An important finding of this study is that by changing the ligand to match the rates of the different steps in a catalytic cycle, substantial improvement may be seen (*e.g.*, the results shown in Scheme 3).§
§We note that several research groups have previously articulated the importance of rate matching in the context of transition metal catalysis. For recent examples, see ref. 37.


In all, the integration of automation and online analytics has enabled a systematic methodology for both optimizing cross-coupling reactions and extracting key mechanistic insights. Given the reaction information that can be gleaned from the simultaneous “smart” study of Pd source, ligand, and continuous variables, it can only be anticipated that future studies consolidating more variables into fewer, more impactful experiments will lend even greater insight into catalytic system design.
