# Supplementary material for: Suzuki–Miyaura cross-coupling optimization enabled by automated feedback
Source: React Chem Eng. 2016 Oct 18;1(6):658–66. doi: 10.1039/c6re00153j (PMC5123644; doi:10.1039/c6re00153j)
Supplement: Supplementary file 1 [file RE-001-C6RE00153J-s001.pdf]

Electronic Supplementary Material (ESI) for Reaction Chemistry & Engineering.
This journal is © The Royal Society of Chemistry 2016

Supporting Information for
"Suzuki-Miyaura Cross-Coupling Optimization Enabled by Automated Feedback"

Brandon J. Reizman[1], Yi-Ming Wang[2], Stephen L. Buchwald[2], and Klavs F. Jensen[1]

[1]-Department of Chemical Engineering
Novartis-MIT Center for Continuous Manufacturing
Massachusetts Institute of Technology
77 Massachusetts Avenue, Cambridge MA 02139 (USA)

[2]-Department of Chemistry
Novartis-MIT Center for Continuous Manufacturing
Massachusetts Institute of Technology
77 Massachusetts Avenue, Cambridge MA 02139 (USA)

Table of Contents

Experimental Setup 4

Optimization Method 12

Algorithm Implementation 17

General Reagent Information 18

General Analytical Information 19

General Procedure for Preparation of Stock Solutions 19

Comparison of Reaction Yield in Batch and in Droplets 19

Experimental Data 21

Yield Estimation 21

Experimental Trajectories 21

Reaction of 1 and 2 (Case I) 22

Reaction of 4 and 2 (Case II) 24

Reaction of 4 and 6 (Case III) 27

Reaction of 8 and 9 (Case IV) 30

Optimization of Ligand Equivalents 33

Time-Course Evolution of 6 35

Reaction of 4 and 12 37

Product Isolation 38

General Procedure for the Synthesis of Authentic Samples of Biaryls 38

3,5-Dimethyl-4-(quinolin-3-yl)isoxazole (3) 38

3,5-Dimethyl-4-(pyridin-3-yl)isoxazole (5) 39

3-(Benzofuran-2-yl)pyridine (7) 39

tert-Butyl-2-(pyridin-2-yl)-1*H*-pyrrole-1-carboxylate (10) 39

References 45

Experimental Setup

A full system diagram is presented in Figure S.1. On-demand reagent sampling was accomplished using an automated liquid handling robot (Gilson GX-271, Gilson, Inc.) controlled with LabVIEW software. A syringe pump (Harvard PhD 2000) with a 100 µL glass syringe was connected to the liquid handler probe (needle) by approximately 50 cm of 500 µm PFA tubing filled with a selected transport fluid. To prepare a droplet, the liquid handler aspirated first a 30 µL volume of inert gas, followed by aliquots of the solvent tetrahydrofuran (THF), the desired precatalyst (and ligand, if applicable), the desired reactants, water, and THF again. To minimize carryover during this process, the liquid handler probe was dipped in a THF wash solution before each reagent aspiration. 35 µL total liquid plus a density correction volume were aspirated. Following sample aspiration, the sample was "stirred" three times in the probe under inert conditions by pulling and pushing with the syringe pump 30 µL volume. All reagents were then transferred into a 6 port-2 way injection valve (Cheminert 10S-0503H, Valco Instruments Co. Inc.) containing a 14-µL sample loop. Switching of the sample loop to the inject position created a 14-µL droplet.

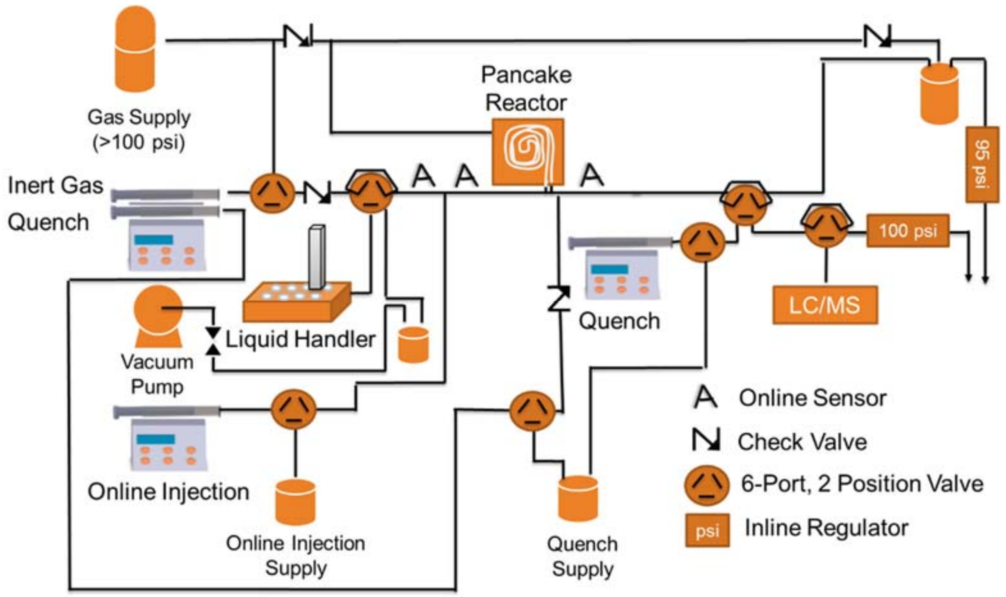

Figure S.1. Schematic of automated flow system for reaction optimization.

As reagent carryover from one droplet to the next had the potential to significantly and adversely affect the accuracy of the optimization, we included in the sampling procedure the preparation of three blank droplets before every on-demand droplet preparation. These served to clean the probe, injection valve, sample loop, and reaction system of any residual material from prior experiments. The compositions of the three blank droplets were, respectively: water, acetone, and THF (the use of the reaction solvent as the final blank droplet was advantageous in case any blank material became deposited on the reactor walls). In sequence, the liquid handler aspirated 20 μL inert gas, followed by 60 μL of each wash solvent with injection. The sample injection valve and sample loop were cleared following every injection by pulling a vacuum for 3 sec on the outlet of the valve. This was accomplished by switching on and off a solenoid 3-way valve (P/N 01540-11, Cole-Parmer Instrument Company, LLC) connecting the house vacuum to a trap connected to the outlet of the valve. Residual liquid was drained from the trap at the end of an optimization. Additionally, a 6-port, 2-way valve (Rheodyne MXP7960-000, IDEX Health & Science LLC) installed on the transfer line between the syringe pump and the probe was used to refill the transfer line with 80 μL fresh transfer fluid after every on-demand droplet preparation. The line was connected through an inline degasser (Agilent G1379B μ-degasser) to a supply tank of transfer fluid (THF). This was found to both help minimize reagent carryover (from the material transferred to the transfer fluid during aspiration and stirring) and reduce the frequency of gas bubble formation in the transfer line. To remove gas bubbles formed from the probe sitting idle, the transfer line purge was repeated three times at the start of any optimization.

As reagents with different densities were sampled by the air displacement method, a correction factor corresponding to the density ratio between the sampled fluid and the transfer fluid was introduced to ensure accurate transfer. The relative aspirated volumes of samples 1 and 2 was expressed as:

$$\frac{\Delta V_1}{\Delta V_2} = \frac{\rho_2}{\rho_1} \tag{1}$$

where ρ_1 and ρ_2 were the densities of two fluids and ΔV_1 and ΔV_2 were the relative volumes sampled by the air displacement method.

The minimum volume of a prepared droplet was restricted both by the sampling accuracy and the dead volume of the sample injection valve. At approximately 0.1% of the syringe volume, the minimum sample accuracy was roughly 100 nL; thus reagent sampling of less than 1 μL implied greater than 10% error in the sample. Generally it was undesirable for quantitation to aspirate reagent samples of less than 3 μL, though given that the target range for optimizations was generally a factor of 5 (for instance 0.5% to 2.5% catalyst) sometimes as little as 2 μL reagent was sampled with the acceptance that at the low end of the optimization this implied 5% error. The total sample volume aspirated needed to fill both the dead volume of the injection valve (estimated as 15-20 μL) and the 14-μL sample loop; hence nominally 35 μL of sample was aspirated. As many as six reagents were mixed in the liquid handler for a single droplet.

The ability to keep samples under inert atmosphere and relatively free of evaporation was an important aspect to our optimization system, particularly for the case of catalyst screening. To provide inertion, we designed a 3D-printed manifold that allowed for an argon blanket to be maintained over air-sensitive reagents. The manifold is depicted in illustration and in application in Figure S.2(a-b). Screw threads on the underside of the manifold allowed vials containing reagents in solution to tighten against 15 mm PTFE-lined red rubber septa (W240594SP, Wheaton). A void space was left open above the vials through which argon at low pressure was supplied. 15 mm PTFE-lined red rubber septa were then inserted above the void space to seal the top of the manifold. The manifold itself was printed by Solid Concepts, Inc., from PEEK HP3. For reactions in THF, the evaporation of THF with such a device was of concern to the accuracy of our method. We found that reducing the flow of argon with a bleed valve helped greatly to reduce evaporation, as did filling the vial closest to the argon inlet with neat THF (such that the atmosphere inside of the manifold would be THF saturated). Nonetheless, we were only able to run experiments for ~48 h before observing losses in accuracy on account of evaporation. We did not observe catalyst deactivation over 48 h on account of the solution stability of this family of precatalysts.

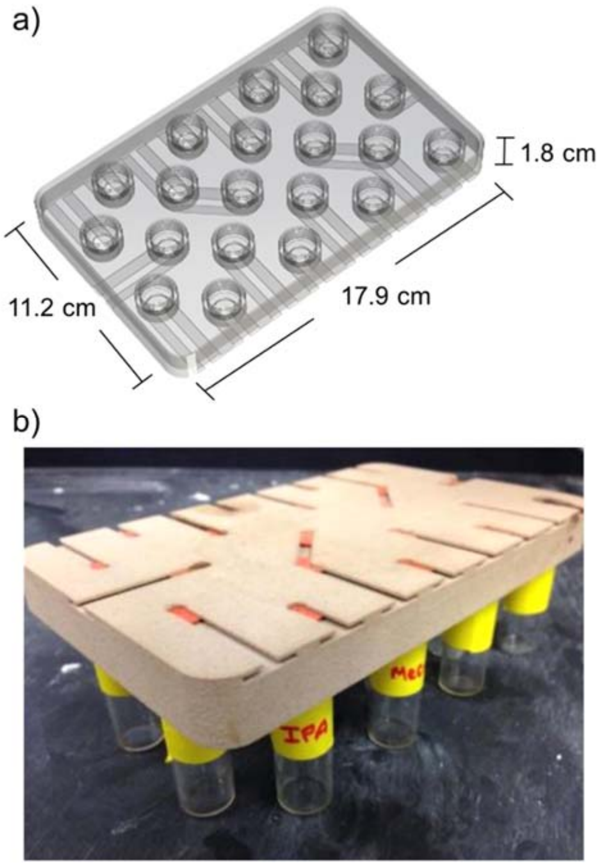

Figure S.2. Septum-sealed inert gas manifold for reagent storage under inert gas atmosphere, (a) SOLIDWORKS rendering and (b) photograph of 3D-printed device.

The use of argon (≥99.997%, Airgas) avoided the solubility and flow rate limitations introduced by perfluorinated carrier solvents. We observed that in the uses of stainless steel, PEEK, and even PFA tubing led to significant wetting for solvents such as THF, which in turn led to degradation of the droplet along the tubing wall. Considerable improvement was observed with 750 μm inner diameter Teflon fluorinated ethylene propylene (FEP) as the tubing material. Droplets were transported through the FEP tubing (Upchurch Scientific) by an 8 μL stainless steel syringe (Harvard Apparatus) containing 6.9 bar (100 psi) argon driven by syringe pump (Harvard PhD 2000). The compressibility of the gas mandated that for steady flow rates to be achieved, constrictions in the flow path had to be limited to no less than 500 μm and the pressure be maintained at or above 6.9 bar. A check valve (Upchurch Scientific) was installed upstream of droplet preparation and injection to dampen the effect of pressure oscillations further. Sample loops and unions upstream of the reactor were made from Teflon to ensure as little carryover as

possible in the system. More consistent gas-liquid flow was observed by use of a 1 mm inner diameter T-junction at the reaction quench. Pressure in the system was controlled at 6.9 bar with an inert gas-regulated Parr bomb, approximately 40 mL in volume. The bomb was drained during refill of the gas and quench syringes by automatically opening a 6-port, 2 way valve (Rheodyne MXT715-000). To minimize gas loss during regular system operation, 6.6 bar of backpressure (5.2 bar and 1.4 bar backpressure regulators, Upchurch Scientific) was applied to the gas vent of the Parr bomb during regular system operation.

For reaction, the FEP tubing was inserted into a "pancake" reactor housing (Figure S.3(a-b)), comprising of an aluminum chuck with a 1.6 mm groove for the tubing, a raised lip with an O-ring, and a sheet of polycarbonate which compressed against the O-ring to allow for pressurization of the reactor to 6.9 bar. With this device, we were able to rapidly heat and cool the reactor tubing between 30 °C and 110 °C and neutralize gas permeation out of the reactor—a factor which accounted for up to a 20% difference in residence time at high temperature. Residence times in the reactor were maintained between 1 min and 10 min (we empirically observed mixing to take place on the order of 1-10 s) at gas flow rates of 15-250 μL/min. A thermocouple was introduced through the gas supply line of the reactor and held in place on the aluminum surface by a thin sheet of polycarbonate and thermal paste. The reactor was heated with four 50 W cartridge heaters (McMaster-Carr Supply Company, two pairs spaced equally on opposite sides of the device). A PID temperature controller (OMEGA CN9412) controlled the reactor temperature. Reaction droplets were not introduced into the system unless the reactor temperature was within 1 °C of the reaction set point temperature. Because acceleration of droplets was observed to occur when trailing droplets entered the heated reactor (a consequence of surface-tension driven thermocapillary flow[1]), blank droplets were not prepared and introduced until a reaction droplet had traversed a full reactor volume in the system (240 μL).

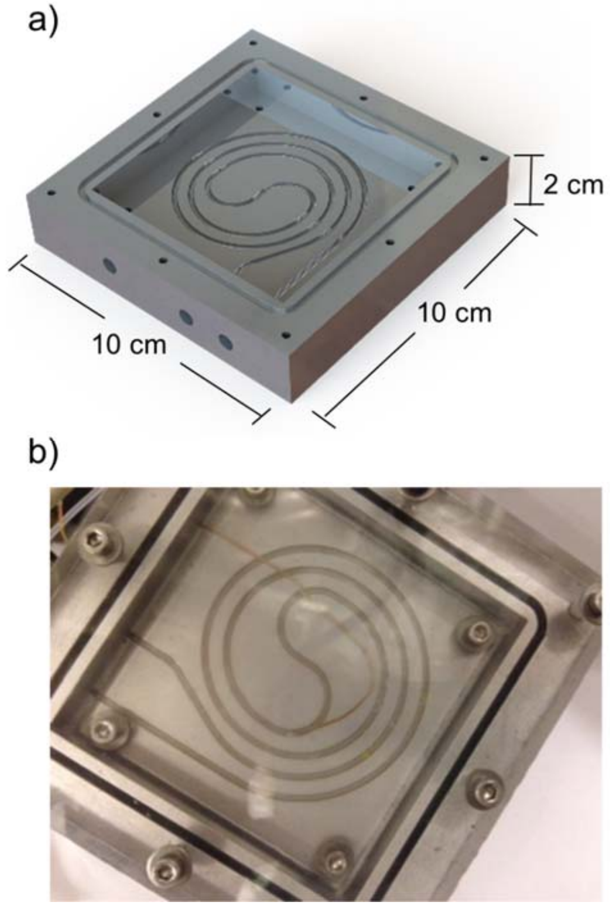

Figure S.3. Pressure-sealed "pancake" reactor comprising a Teflon tube in an aluminum housing, (a) SOLIDWORKS rendering and (b) photograph of packaged device with polycarbonate cover, FEP tubing, and thermocouple.

The online injection reagent (1.66 M DBU in THF) was stored under an inert atmosphere and sampled by a 250 μL glass syringe (Gastight, Hamilton Company) syringe driven by a syringe pump (Harvard PhD 2000). A 6-port, 2-way valve (Rheodyne MXP7960-000) was installed on the line to allow switching between refill of the syringe and online injection. Under normal continuous flow conditions, the valve was exclusively in the online injection position. Approximately 1 m of 250 μm inner diameter PFA tubing connected the valve to a T-junction (500 μm ID Teflon, Upchurch Scientific), which intersected the main system 6 cm before the reactor inlet. As a droplet passed through the T-junction, the syringe pump infused 3.5 μL base solution into the droplet. The flow rate of the injection was chosen such that the volume was infused while 80% of the droplet passed through the T-junction. Refractive index sensors (EE-SPX613, Omron Corporation) were attached to the Teflon tubing before and after the T-junction to correctly time the online reagent

injection and to verify that the droplet volume was within an acceptable tolerance (±4.0 μL) following the online injection. This was intended as a verification that no gas bubbles were introduced into the droplet and that the droplet had not broken apart upstream.

Leaks and gas bubbles were consistent inhibitors of reliable online injection. To ensure accuracy to 100-250 nL, the only chemically-suitable commercial options available for reagent dosing were glass syringes, which were found to leak over the course of a few optimizations with exposure to 6.9 bar pressure. The connection of the glass syringe to Teflon tubing was also often a source of leaks. We found the use of female Luer connectors sold by Upchurch Scientific to be most leak resistant, in comparison to the use of ¼-28-Luer adapters. Use of the 6-port, 2-way valve for refilling was mandatory compared to a 3-way solenoid valve because of the smaller dead volume and better pressure tolerance. To avoid the introduction of gas bubbles into the injection line, the entire refill line was purged when new stock solutions were introduced by detaching and refilling the injection syringe. Naturally it was desirable to make the connection between the syringe and the T-junction as short as possible. By using a Teflon T-junction, less sticking and carryover of reagents were observed. A 500 μm T-junction was also required to prevent injected material or segments of droplets from becoming trapped in the swept volume of the T.

Downstream of the reactor, droplets were quenched at room temperature with a continuously flowing solution of 50/50 acetone/water delivered *via* syringe pump (Harvard Apparatus PhD 2000 with 8 mL Harvard stainless steel syringe) through a T-junction (1 mm ID Teflon, Upchurch Scientific). A third refractive index sensor was used downstream of the quench to time the HPLC sampling accurately. Following sampling with a 30 μL sample loop in a 6-port, 2-way valve (Gilson Valvemate II), the sample was transported *via* syringe pump (Harvard Apparatus PhD 2000 with 1 mL Hamilton Gastight syringe) containing 50/50 acetone/water to a second 6-port, 2-way valve (Agilent G1158A) with a 1 μL PEEK sample loop. An LC/MS (Agilent G1312B binary pump, G1329B ALS, G1316A column compartment, G1365C multi-wavelength detector, 6120 quadrupole MS) method was remotely started with LabVIEW software. The LC flow rate increased from 0.5 mL/min (standby mode) to 3.5 mL/min (required for method), and the sample was injected into the HPLC after 15 s. The sample passed through a 0.5 μm filter (Upchurch Scientific), then was heated to 40 °C, passed through a T-junction (250 μm stainless steel Valco),

and split by pressure difference between a 1.8 μm particle diameter column (Agilent Zorbax SB-C18 2.1 x 50 mm) and a 4.6 μm particle diameter column (Agilent Zorbax SB-C18 2.1 x 50 mm). The sample from the 1.8 μm particle diameter column was detected by UV and passed to the MS. A suitable HPLC method was found to be 9 min, which included a gradient ramp from 95/5 water/acetonitrile + 1% formic acid to 0/100 water/acetonitrile + 1% formic acid to 95/5 water/acetonitrile + 1% formic acid. Following UV analysis, the spectral baseline found by subtraction of a reference and ChemStation outputted an Excel data file which was retrieved by LabVIEW (National Instruments, ver. 8.6). The product yield was calculated in MATLAB (The MathWorks, Inc., ver. R2011a).

Valve manipulation, HPLC method initiation, and analog input communication with the refractive index sensors were accomplished using a Compact FieldPoint controller from National Instruments (cFP-2020, cFP-RLY-425, cFP-AI-110). The entire system including pumps, liquid handler, temperature control, refractive index monitoring, valving, remote triggering of the HPLC gradient, and MATLAB optimization was controlled with LabVIEW. With the exception of drivers, which were obtained from suppliers or from National Instruments, the software in LabVIEW was written completely in-house for the purpose of on-demand reaction screening. The LabVIEW routine comprised a central VI that executed simultaneous loops for flow rate manipulation, reaction preparation, temperature control, online monitoring, HPLC sampling, HPLC analysis, and optimization. Data for individual droplet experiments were recorded in a single matrix in MATLAB. These data were recorded every 20 s in the form of text files documenting information about each droplet experiment (concentrations, reagents, set temperature, and set reaction time), status of the experiments (time spent by droplet in system and analysis and objective function value), system information (current flow and temperature conditions), and summaries of data analysis. Termination criteria were determined with MATLAB.

Optimization Method

To control and optimize the system, we developed LabVIEW virtual instruments and MATLAB functions that given a set of discrete variables (precatalysts, ligands) and continuous variable ranges (temperature, reaction time, catalyst loading) and online HPLC data, modeled the reaction yield as a function of variable contributions and iteratively proposed new experiments to study using optimal DoE.[2] Variables were randomized and all discrete variables were treated as yes/no decisions; shared catalyst attributes did not factor into the algorithm's calculations.

To identify a proper scaling for the factors in our response surface methods, we began with consideration of the generalized chemical reaction A + B → R over N_{cv} continuous variables and N_{dv} discrete variables (for instance candidate catalysts for the synthesis of R). A general formulation of the mixed-integer nonlinear programming (MINLP) for optimization of the chemical system was:

$$\begin{aligned} &\max_{\{\mathbf{x},\mathbf{y}\}} f(\mathbf{x},\mathbf{y}) \\ &\text{s.t. } g(\mathbf{x},\mathbf{y}) \le 0 \\ &\sum y_i = 1 \\ &x_j \in [-1,1] \quad \text{for } j = 1,\dots,N_{cv} \\ &y_i \in \{0,1\} \quad \text{for } i = 1,\dots,N_{dv} \end{aligned} \tag{2}$$

where $f(\mathbf{x},\mathbf{y})$ was the turnover number (TON) of the reaction—defined as moles product per mole of catalyst—and the constraint $g(\mathbf{x},\mathbf{y})$ was a constraint on the minimum yield (C_R/C_{A0}) at the optimum:

$$g(\mathbf{x},\mathbf{y}) = \gamma Y(\mathbf{x}',\mathbf{y}') - C_R / C_{A0}, \text{ where } Y(\mathbf{x}',\mathbf{y}') = \max_{\{\mathbf{x},\mathbf{y}\}} C_R / C_{A0} \quad \begin{aligned} &\text{s.t. } \sum y_i = 1 \\ &x_j \in [-1,1] \quad \text{for } j = 1,\dots,N_{cv} \\ &y_i \in \{0,1\} \quad \text{for } i = 1,\dots,N_{dv} \end{aligned} \tag{3}$$

The parameter γ was adjustable in the range [0,1], with the choice of $\gamma = 1$ implying maximization with respect to yield and $\gamma = 0$ implying unconstrained maximization of TON.

For the generalized bimolecular reaction A + B → R with constant $k_R = k_R$(catalyst type, C_{cat}, T), the production rate C_R/t_{res} was assumed to scale on the order of:

$$\frac{C_R}{t_{res}} \propto k_R\left(\text{catalyst}, C_{cat}, T\right) C_{A0}^m C_{B0}^n \tag{4}$$

Although this scaling would not apply universally to the chemical kinetics of a catalytic system, we assumed that near an optimum a single apparent rate-limiting step of this form would dominate. For simplicity, it was then assumed that k_R(catalyst type, C_{cat}, T) could be separated into Arrhenius and catalyst-specific terms:

$$k_R\left(\text{catalyst}, C_{cat}, T\right) \propto \left(C_{cat}^p A_i e^{-E_{Ai}/RT}\right)\left(A_R e^{-E_{AR}/RT}\right) \tag{5}$$

giving an assumed scaling for C_R of:

$$C_R \propto A_i A_R e^{-(E_{Ai}+E_{AR})/RT} C_{A0}^m C_{B0}^n C_{cat}^p t_{res} \tag{6}$$

Taking the logarithm of all factors produced the linear relation:

$$\ln\left(C_R\right) \propto \ln\left(A_i\right) + \ln\left(A_R\right) - \frac{E_{Ai}}{R}\left(\frac{1}{T}\right) - \frac{E_{AR}}{R}\left(\frac{1}{T}\right) + m\ln\left(C_{A0}\right) + n\ln\left(C_{B0}\right) + p\ln\left(C_{cat}\right) + \ln\left(t_{res}\right) \tag{7}$$

This introduced a set of continuous factors to vary for each discrete variable: T^{-1}, $\ln(C_{i0})$, $\ln(C_{cat})$, and $\ln(t_{res})$. Naturally the assumptions leading to Equation 7 ignored the possibility of more complex kinetics, such as a Langmuir-Hinshelwood mechanism,[3] across the full experimental space or the change in rate as the starting reagents A and B were consumed. To correct for inaccuracies in our assumed scaling, additional coefficients were introduced to weigh the $\ln(t_{res})$ term and account for interactions and quadratic functionality among all continuous variables. The final response surface model to fit was of the form:

$$\hat{b} = \sum_{i=1}^{N_{cat}}\left(c_i y_i + a_{i1} y_i x_1\right) + \sum_{j=2}^{N_{cv}} c'_j x_j + \sum_{j=1}^{N_{cv}} \sum_{j'=j}^{N_{cv}} a'_{jj'} x_j x_{j'} \tag{8}$$

where $\hat{b}$ was the response value and a_{i1}, $a'_{jj'}$, c_i, and c'_j, were coefficients to fit. x_1 was assigned to the scaled T^{-1} factor.

Optimal coefficients $\boldsymbol{\theta}$ for the response surface model were found by weighted least-squares regression of the scaled experiments $\mathbf{X} = [\mathbf{x}\ \mathbf{y}]$ (including the linear, interaction, and quadratic terms of discrete and continuous variables in Equation 8) and the vector of measured responses $\mathbf{b}$. We found empirically that the use of a weighting matrix $\mathbf{W}$ which biased the regression to most closely fit the response surface at points where the yield of R was greatest gave the most accurate optimization results. Convenient choices of $\mathbf{W}$ were the yield or TON, giving:

$$\boldsymbol{\theta} = \left(\mathbf{X}^T\mathbf{W}\mathbf{X}\right)^{-1}\mathbf{X}^T\mathbf{W}\mathbf{b};\ \mathbf{W} = \begin{bmatrix} C_{R1}/C_{A01} & 0 & \cdots & 0 \\ 0 & C_{R2}/C_{A02} & & \vdots \\ \vdots & & \ddots & 0 \\ 0 & \cdots & 0 & C_{RN_{expts}}/C_{A0N_{expts}} \end{bmatrix} \text{ or } \begin{bmatrix} C_{R1}/C_{cat1} & 0 & \cdots & 0 \\ 0 & C_{R2}/C_{cat2} & & \vdots \\ \vdots & & \ddots & 0 \\ 0 & \cdots & 0 & C_{RN_{expts}}/C_{catN_{expts}} \end{bmatrix} \quad (9)$$

Optima for the each discrete variable were identified by converting Equation 8 to quadratic form and populating the matrices $\mathbf{A}$ and $\mathbf{c}$ with the optimal parameters $\boldsymbol{\theta}$:

$$\begin{aligned} J_i^* = \max_{[\mathbf{x}\ \ \mathbf{y}]} \hat{b} &= [\mathbf{x}\ \ \mathbf{y}]\mathbf{A}[\mathbf{x}\ \ \mathbf{y}]^T + \mathbf{c}[\mathbf{x}\ \ \mathbf{y}]^T \\ s.t.\ \ln\gamma &+ \ln\left[Y(\mathbf{x}',\mathbf{y}')\right] - \left[\hat{b} + \ln\left(C_{cat}/C_{A0}\right)\right] \le 0 \\ x_j &\in [-1,1] \quad \text{for } j = 1,\ldots,N_{cv} \\ y_i &= 1 \\ y_k &= 0 \quad \text{if } k \ne i \end{aligned} \quad (10)$$

Calculation of $\ln[Y(\mathbf{x}',\mathbf{y}')]$ was performed using the same quadratic model for $\hat{b}$, with linear rescaling with respect to $\ln(C_{cat}/C_{A0})$. The overall maximum, J^*, corresponded to the maximum over all J_i^*.

To calculate the uncertainty on J^*, the prediction covariance $V_{\hat{B}}$ was estimated as:[4]

$$V_{\hat{B}} = [\mathbf{x}^*\ \ \mathbf{y}^*]\left(\mathbf{X}^T V_B^{-1}\mathbf{X}\right)^{-1}[\mathbf{x}^*\ \ \mathbf{y}^*]^T \quad (11)$$

With many experiments at or near the optimum, an estimate for the scalar response covariance V_B could have been obtained from the squared sum of residuals. However with very few experiments,

we observed significant bias in a squared sum of residuals estimate of V_B. To reduce the amount of bias introduced by the manner in which experiments were being selected, a jackknife resampling strategy was employed to generate the scalars V_{Bu}, which were the response covariance values estimated with experiment u removed from the data set:[5]

$$V_{B_u} = \frac{\left(\boldsymbol{\theta}_u{}^T \mathbf{X}'_u - \mathbf{b}_u\right)^T \left(\boldsymbol{\theta}_u{}^T \mathbf{X}'_u - \mathbf{b}_u\right)}{N_{expts} - N_{params} - 1} \tag{12}$$

$\mathbf{X}'_u$, $\mathbf{b}_u$, and $\boldsymbol{\theta}_u$ were the matrix of scaled experimental conditions, vector of responses, and best-fit response surface parameters calculated excluding experiment u, respectively. An overall estimate of V_B was then found by:

$$V_B = \frac{N_{expts}}{N_{expts} - 1} \sum_{u=1}^{N_{expts}} \left[V_{B_u}^{1/2} - \frac{1}{N_{\text{expts}}} \sum_{u=1}^{N_{expts}} V_{B_u}^{1/2} \right]^2 \tag{13}$$

and assumed to be uniform across all response surfaces. We assumed that as the algorithm advanced and conducted more experiments closer to the predicted optima, V_B became more representative of the covariance near [**x*** **y***].

Given an estimate for the response covariance, a lower bound on J^* was found from a Student's t-distribution and $V_{\hat{B}}$ evaluated at the optimum:[4]

$$J_-^* = J^* - \left(V_{\hat{B}}\right)^{1/2} \left(t_{1-\alpha, \nu = N_{expts} - N_{params}}\right) \tag{14}$$

α was chosen before experimentation as 0.05, corresponding to a 95% one-sided confidence level on the lower bound of J^*. For the least optimal discrete variable, a paired 2-sample t-test at 95% confidence revealed whether J_i^* was significantly less than the overall optimum J^*:

$$\text{Null Hypothesis: } H_0 = J^* - \min\left(J_i^*\right) = 0$$
$$H_a = J^* - \min\left(J_i^*\right) > 0$$
$$t_{stat} = \frac{J^* - \min\left(J_i^*\right)}{V_{\hat{B}}^{1/2}} > t_{crit} = t_{1-\alpha,\nu=N_{expts}-N_{params}}$$
$$J^* - \left(V_{\hat{B}}^{1/2}\right)\left(t_{1-\alpha_{conf},\nu=N_{expts}-N_{params}}\right) > \min\left(J_i^*\right)$$
$$J_-^* > \min\left(J_i^*\right) \tag{15}$$

This assumed a constant $V_{\hat{B}}$ for both discrete variable optima**.** Rejection of the null hypothesis resulted in fathoming of discrete variable i from the current optimization step, and response surfaces were recalculated excluding y_i and any data points associated with variable i from the model (though in instances where the number of candidate experiments was less than $N_{params} + 1$, it was possible to leave variable i and associated data points in the model to advance the optimization). This process proceeded in a loop until all values of J_i* exceeded J_-*. The remaining discrete variables comprised the experimental set for which a new iteration of experiments was generated.

To accelerate reduction of the discrete variable space and simultaneously maximize the continuous variable information gained per experiment, new experiments were generated using a modified G-optimality criterion:

$$G_i = \min_{[\mathbf{x}\ \ \mathbf{y}]} \left[\mathbf{x}_i * \quad \mathbf{y}_i *\right]\left(\mathbf{X}_1^T V_B^{-1} \mathbf{X}_1\right)^{-1}\left[\mathbf{x}_i * \quad \mathbf{y}_i *\right]^T + \left[\mathbf{x}_i' \quad \mathbf{y}_i'\right]\left(\mathbf{X}_1^T V_B^{-1} \mathbf{X}_1\right)^{-1}\left[\mathbf{x}_i' \quad \mathbf{y}_i'\right]^T \tag{16}$$
$$s.t.\ x_j \in [-1,1]$$
$$y_i = 1$$
$$y_k = 0 \text{ if } i \neq k$$

$\mathbf{X}_1$ was the matrix **X** augmented to include the candidate experiment [**x y**]. Equal weighting was assigned to minimizing the error in the yield optimum and to minimizing the error in the constrained TON optimum. [$\mathbf{x}_i$* $\mathbf{y}_i$*] was supplied as an initial guess to a sequential quadratic programming (SQP) optimizer to generate G_i and the new G-optimal experimental conditions.

These experiments were then executed, and new response surfaces and estimates for J_i* were estimated accordingly.

To achieve convergence, we specified that linear improvement had to be observed both in the predicted optimal TON (exp(J*)) and in the lower bound on the optimal TON (exp(J_-*)) to within 2% of the optimal value:

$$
\begin{aligned}
&\text{let } J_q^* \text{ be the the optimum through iteration } q \\
&J_{pred,q}^* = \frac{N_{expts} \text{ between } q\text{ - 2 and } q}{N_{expts} \text{ between } q\text{ - 2 and } q\text{ -1}}\left(J_{q-2}^* - J_{q-1}^*\right) + J_{q-2}^* \\
&\text{if } \frac{\left|J_{pred,q}^* - J_q^*\right|}{J_q^*} \text{ and } \frac{\left|J_{pred,q-1}^* - J_{q-1}^*\right|}{J_{q-1}^*} < 0.02 \\
&\text{and } \frac{\left|J_{-pred,q}^* - J_{-q}^*\right|}{J_{-q}^*} \text{ and } \frac{\left|J_{-pred,q-1}^* - J_{-q-1}^*\right|}{J_{-q-1}^*} < 0.02, \text{ terminate}
\end{aligned}
\tag{17}
$$

Importantly, this criterion was independent of the number of remaining unfathomed solvents (meaning multiple optima could be obtained within the convergence tolerance) and independent of the scaling of f. Alternatively to avoid adverse effects of solvent evaporation, the method also terminated when the total number of experiments exceeded 96.

Algorithm Implementation

Full catalyst-ligand optimization studies commenced with a randomized 16-experiment fractional factorial design, followed by a second refined 16-experiment fractional factorial design. The ligand equivalent optimization study commenced with a randomized 12-experiment fractional factorial design, followed by a second refined 12-experiment fractional factorial design. To prevent a loss in accuracy from solvent evaporation, optimization studies were terminated at a maximum of 96 experiments, regardless of whether the termination criteria presented above had been satisfied. Optimization routines were executed in MATLAB. The constraint parameter γ was chosen as 0.90. The reaction TON was chosen as the weighting factor in the least squares regression for all examples except for the optimization of 2-chloropyridine (**8**) and *N*-boc-2-pyrroleboronic acid (**9**), where yield was used for the weighting.

Following initialization, response surfaces were iteratively developed for candidate discrete variables and discrete-variable-specific optima were predicted based on the response surfaces. Using a procedure akin to branch and bound, discrete variables whose performance was worse than the lower bound on the maximum of the leading discrete variable were fathomed from that iteration of the optimization, and response surfaces were recalculated using only experimental data from the remaining candidate discrete variables. Once a candidate set of discrete variables was determined, new experiments were chosen by G-optimality[2] and the procedure repeated until convergence to the final optimum.

To ensure continuous operation and minimize downtime, new optimal experimental conditions were calculated one experiment before the complete data set for a given iteration was collected. It was also a very realistic possibility in practice that zero product yield and TON would be observed, which in this algorithm's logarithmic coordinate system would have produced an undefined objective value. Through simulations, we found that assigning a yield of 0.1% to cases where the yield was in truth zero imposed sufficient penalty on the optimization method, yet did not interfere with prediction of the maximum. The detection limit by HPLC was also chosen to be within an order of magnitude of 0.1% of the maximum in experiments. Optimal experiments were grouped by temperature and randomized at each optimization iteration to minimize both experimental bias and the time required for temperature re-equilibration.

General Reagent Information

THF was dried and deoxygenated by passing through packed columns of neutral alumina and copper(II) oxide under a positive pressure of argon. Water was deoxygenated by sonicating under vacuum and backfilling with argon, (this process was repeated a total of three times). Aryl halides were purified by filtration through neutral alumina. The precatalyst-ligand complexes used in this study were prepared following the procedure reported by Bruno *et al*.[5] All other reagents were purchased from Oakwood, Combi-Blocks, Alfa Aesar, or Sigma Aldrich and used as received. Flash chromatography was performed with *SiliCycle SiliaFlash*® F60 silica gel.

General Analytical Information

Compounds were characterized by ^{1}H NMR and ^{13}C NMR. Copies of the ^{1}H NMR and ^{13}C NMR spectra can be found at the end of the Supporting Information. ^{1}H and ^{13}C NMR spectra were recorded on a Bruker 400 MHz or 600 MHz instrument. All ^{1}H NMR data are reported in δ units, parts per million (ppm), and were measured relative to the residual proton signal in the deuterated solvent at 7.26 ppm ($CDCl_3$). All ^{13}C NMR spectra are ^{1}H decoupled and reported in ppm relative to the solvent signal at 77.16 ppm ($CDCl_3$). Thin-layer chromatography (TLC) was performed on *Silicycle* 250 μm silica gel plates. Compounds were visualized by irradiation with UV light, or stained with I_2/SiO_2, $KMnO_4$, or phosphomolybdic acid (PMA). Yields refer to isolated compounds, unless otherwise indicated.

General Procedure for Preparation of Stock Solutions

The reagent solutions were freshly prepared under ambient conditions but stored under argon for each optimization or kinetic parameter ramp. 7 mL vials and tapered 2 mL vials were initially sealed and inerted with nitrogen. In a 5 mL volumetric flask, aryl halide (7.0 mmol) and naphthalene (2.5 mmol, as the internal standard) were diluted with THF (1.4 M aryl halide, 0.50 M naphthalene) and then transferred to a 7 mL vial. A separate 5 mL volumetric flask was charged with boronic acid or boronic pinacol ester (5.0 mmol), diluted with THF (1.0 M) and transferred to a 7 mL vial. Separate vials of precatalyst solutions were prepared by adding the solid precatalyst (0.036 mmol) to a tapered 2 mL vial, then diluting with THF (2 mL, 0.018 M). A 10 mL volumetric flask was charged with DBU (2.5 g, 16 mmol), diluted with THF (1.6 M) and transferred to a 20 mL scintillation vial.

Comparison of Reaction Yield in Batch and in Droplets

To validate the scalability of our method, we compared the results in batch of the Suzuki-Miyaura cross-coupling reaction in Scheme S.1 to results from the segmented flow system. The batch procedure was as follows. 1 M stock solution of DBU in 1:1 (v/v) THF:H_2O. To a 7 mL vial were charged 2-chloropyridine (**8**, 142.0 mg, 1.25 mmol), *N*-boc-2-pyrroleboronic acid (**9**, 430.0

mg, 2.0 mmol) **L1-P1** (22.6 mg, 0.027 mmol), naphthalene (24.4 mg, 0.19 mmol) as the internal standard, and THF (2.0 mL). A 400 µL aliquot of this reaction mixture was transferred to a 7 mL vial, then THF (600 µL) and DBU stock solution (500 µL, 0.5 mmol, 1 M) were added. The reaction was stirred under argon at 60 ºC for 10 min, then quenched with a 1:1 solution of CH_3CN and water, and a small aliquot was analyzed by LC/MS. The product **10** was isolated by column chromatography in 57% yield.

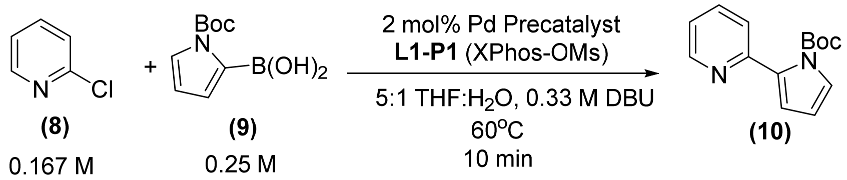

Scheme S.1. Suzuki-Miyaura cross-coupling of 2-chloropyridine and *N*-boc-2-pyrroleboronic acid.

The reaction in Scheme S.1 was repeated in the flow reaction system. The stock solutions were prepared according to the General Procedure using **8** (135.5 mg), naphthalene (47.3 mg), **9** (419.8 mg), and **L1-P1 (**94.0 mg). 14 µL droplets were prepared by sampling the reagent solution, THF, and water in a 1:0.875:0.625 ratio. The base solution (1 M DBU in THF) was introduced online in a 1:2 ratio with the droplets, forming 21 µL reaction segments. The segments reacted at 60 ºC for 10 min with argon carrier gas and were analyzed by LC/MS. Figure S.4 shows the agreement observed between batch and droplet flow yields.

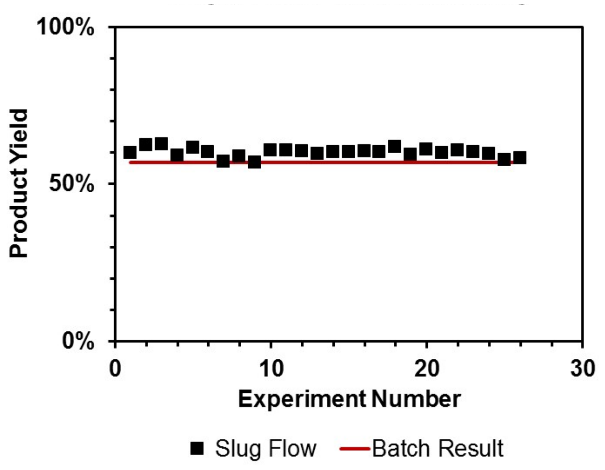

Figure S.4. Comparison of droplet flow yields to batch yield for the reaction of 2-chloropyridine and *N*-boc-2-pyrroleboronic acid with DBU base.

Experimental Data

Yield Estimation

Online HPLC yields were estimated *via* best-fit calibration from a least-squares regression of the measured output concentration of aryl halide and product. The least-squares regression was constrained to enforce mole balance closure on the amount of product formed in reaction. Once optimal conditions were established, products were synthesized, isolated, and characterized in batch.

Experimental Trajectories

Trajectories for experimental case studies I-IV are illustrated in Figure S.5(a-d).

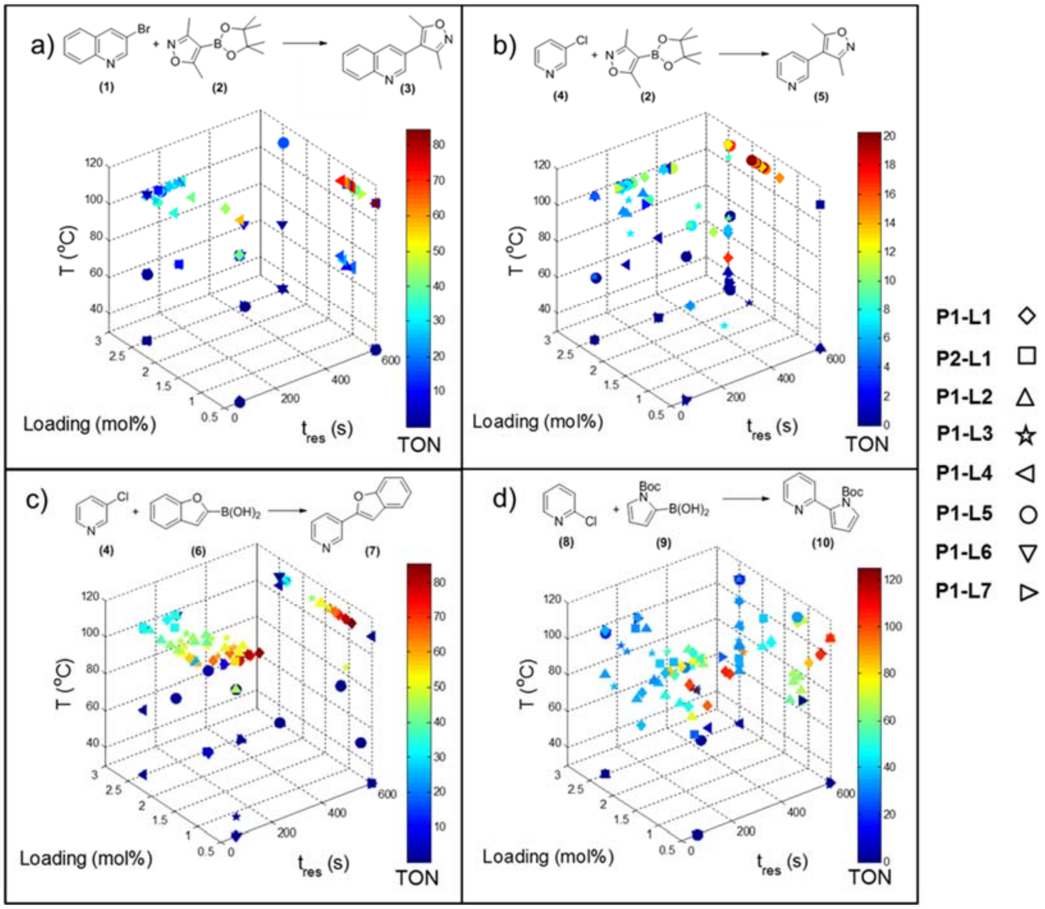

Figure S.5. Automated optimization trajectories for the reactions of (a) 1 and 2, (b) 4 and 2, (c) 4 and 6, and (d) 8 and 9.

Reaction of 1 and 2 (Case I)

Following the General Procedure using 3-bromoquinoline (**1,** 1450.2 mg), naphthalene (340.3 mg), (3,5-dimethylisoxazol-4-yl)boronic acid pinacol ester (**2**, 1152.1 mg). The mass for each precatalyst was: 29.6 mg **P1-L1**, 28.2 mg **P2-L1**, 28.6 mg **P1-L2**, 30.0 mg **P1-L3**, 33.2 mg **P1-L4**, 25.2 mg **P1-L5**, 21.5 mg **P1-L6**, and 20.9 mg **P1-L7**. Solution volumes were automatically sampled to achieve 0.167 M aryl halide, 0.250 M boronic acid pinacol ester, 0.333 M DBU, 0.000835-0.004175 M precatalyst-ligand, and a 5:1 THF-water ratio in the reacting droplets. The product 3,5-dimethyl-4-(quinolin-3-yl)isoxazole (**3**) was detected by UV at 340 nm. Reaction data are presented in Table S.1. Optimization results are presented in Table S.2.

Table S.1. Experimental data for reaction optimization of 1 and 2.

Experiment	Catalyst	t_{res} (s)	T (ºC)	Cat. Loading (mol%)	TON	Yield (%)
1	P1-L3	600.0	30.0	0.498	1.1	0.6
2	P1-L6	600.0	30.0	2.515	0.2	0.6
3	P1-L4	60.0	30.0	2.508	0.2	0.6
4	P1-L1	60.0	30.0	0.513	1.1	0.6
5	P1-L2	600.0	30.0	2.513	0.2	0.6
6	P1-L5	60.0	30.0	0.508	1.1	0.6
7	P1-L7	600.0	30.0	0.506	1.1	0.6
8	P2-L1	60.0	30.0	2.509	0.2	0.6
9	P2-L1	600.0	110.0	0.496	8.5	4.3
10	P1-L4	600.0	110.0	0.512	84.7	43.4
11	P1-L6	60.0	110.0	0.498	1.1	0.6
12	P1-L1	600.0	110.0	2.509	24.0	60.2
13	P1-L5	600.0	110.0	2.512	16.7	42.0
14	P1-L7	60.0	110.0	2.499	33.8	84.6
15	P1-L2	60.0	110.0	0.508	16.9	8.5
16	P1-L3	60.0	110.0	2.489	21.8	54.4
17	P1-L7	189.7	65.3	1.123	0.5	0.6
18	P1-L1	189.7	65.3	1.106	22.5	24.9
19	P1-L6	600.0	65.3	2.515	0.2	0.6
20	P2-L1	189.7	65.3	2.509	8.4	21.1
21	P1-L4	189.7	65.3	2.508	21.2	53.1
22	P1-L5	189.7	65.3	1.106	0.5	0.6
23	P1-L3	600.0	65.3	1.106	5.1	5.6
24	P1-L2	600.0	65.3	1.129	6.7	7.5
25	P1-L4	600.0	110.0	1.106	82.6	91.3
26	P1-L1	600.0	110.0	2.509	27.4	68.6
27	P1-L2	189.7	110.0	2.513	21.1	52.9
28	P1-L6	189.7	110.0	1.127	6.6	7.4
29	P1-L7	600.0	110.0	2.499	30.0	75.0
30	P1-L3	189.7	110.0	2.489	21.6	53.8
31	P1-L5	600.0	110.0	2.512	18.9	47.6
32	P2-L1	600.0	110.0	1.131	15.0	17.0
33	P1-L4	600.0	30.0	0.512	1.1	0.6
34	P1-L5	600.0	30.0	0.508	1.1	0.6

Table S.1. (cont.) Experimental data for reaction optimization of 1 and 2.

Experiment	Catalyst	t_{res} (s)	T (ºC)	Cat. Loading (mol%)	TON	Yield (%)
35	P1-L1	600.0	30.0	0.513	1.1	0.6
36	P2-L1	600.0	30.0	0.496	1.1	0.6
37	P1-L2	60.0	110.0	2.513	16.6	41.9
38	P1-L3	60.0	110.0	2.489	31.2	77.5
39	P1-L6	60.0	110.0	2.515	3.4	8.5
40	P1-L7	60.0	110.0	2.499	34.2	85.4
41	P1-L4	60.0	67.5	2.508	13.4	33.4
42	P1-L5	60.0	66.7	2.512	0.2	0.6
43	P1-L3	60.0	67.0	2.489	9.8	24.4
44	P1-L7	60.0	66.8	2.499	1.4	3.6
45	P1-L1	60.0	66.3	2.509	4.9	12.4
46	P2-L1	60.0	67.0	2.509	4.5	11.2
47	P1-L7	155.6	110.0	2.499	32.5	81.3
48	P1-L5	109.3	110.0	2.482	15.8	39.1
49	P2-L1	104.5	110.0	2.509	11.3	28.4
50	P1-L1	109.2	110.0	2.482	29.8	74.1
51	P1-L3	166.5	110.0	2.489	24.5	60.9
52	P1-L4	60.0	110.0	0.512	20.1	10.3
53	P1-L7	60.0	110.0	0.506	1.1	0.6
54	P1-L3	60.0	110.0	0.498	43.7	21.7
55	P1-L1	60.0	110.0	0.513	40.0	20.5
56	P1-L4	60.0	110.0	0.512	17.0	8.7
57	P1-L3	600.0	110.0	0.968	24.5	23.7
58	P1-L1	600.0	110.0	0.971	42.6	41.4
59	P1-L5	600.0	110.0	0.957	40.6	38.8
60	P1-L4	600.0	110.0	1.268	72.4	91.8
61	P1-L7	600.0	110.0	0.814	46.6	38.0
62	P1-L4	161.7	110.0	2.104	39.9	84.0
63	P1-L1	146.1	110.0	2.509	27.7	69.6
64	P1-L3	185.5	110.0	2.489	25.2	62.8
65	P1-L7	176.5	110.0	2.499	27.5	68.6
66	P1-L1	60.0	110.0	2.266	37.1	83.9
67	P1-L4	60.0	110.0	1.915	34.4	65.7
68	P1-L7	60.0	110.0	2.303	35.0	80.7
69	P1-L3	60.0	110.0	2.323	27.7	64.4
70	P1-L4	600.0	110.0	2.508	30.8	82.1
71	P1-L4	600.0	110.0	2.508	31.9	80.0
72	P1-L4	600.0	67.3	1.214	24.4	29.6
73	P1-L4	600.0	68.7	1.268	16.1	20.4
74	P1-L4	199.3	110.0	1.241	57.8	71.6
75	P1-L1	202.8	110.0	1.592	44.6	70.9
76	P1-L4	600.0	110.0	1.160	65.1	75.5
77	P1-L4	600.0	110.0	1.160	71.4	82.7
78	P1-L4	600.0	110.0	1.106	65.3	72.2
79	P1-L4	600.0	110.0	1.106	83.9	92.8
80	P1-L4	600.0	110.0	1.187	60.7	72.1
81	P1-L4	600.0	110.0	1.106	73.0	80.7
82	P1-L4	600.0	66.3	0.998	20.2	20.1
83	P1-L4	600.0	67.6	0.998	18.0	17.9
84	P1-L4	600.0	110.0	1.241	70.5	87.5
85	P1-L4	600.0	110.0	1.268	63.2	80.2
86	P1-L4	600.0	110.0	1.187	65.8	78.1

Table S.1. (cont.) Experimental data for reaction optimization of 1 and 2.

Experiment	Catalyst	t_{res} (s)	T (ºC)	Cat. Loading (mol%)	TON	Yield (%)
87	P1-L4	189.1	110.0	2.508	39.4	98.7
88	P1-L4	600.0	110.0	1.268	65.6	83.1
89	P1-L4	199.8	110.0	2.508	33.5	83.9
90	P1-L4	199.6	110.0	2.508	33.2	83.2
91	P1-L4	600.0	110.0	1.241	62.0	76.9
92	P1-L4	600.0	110.0	1.025	73.3	75.0
93	P1-L4	600.0	110.0	1.079	81.2	87.6
94	P1-L4	600.0	110.0	1.133	67.0	75.9
95	P1-L4	600.0	110.0	1.052	65.4	68.7
96	P1-L4	600.0	110.0	1.106	71.2	78.7

Table S.2. Optimal yield and TON conditions for optimization of 1 and 2.

	Yield Maximum				TON Maximum			
Catalyst	t_{res} (s)	T (ºC)	Cat. Loading (mol%)	Yield (%)	t_{res} (s)	T (ºC)	Cat. Loading (mol%)	TON
P1-L1	242.8	110.0	2.152	78.7	242.8	110.0	2.152	36.5
P2-L1	96.0	110.0	2.500	20.3	96.0	110.0	2.500	8.1
P1-L2	82.0	110.0	2.500	46.2	82.0	110.0	2.500	18.5
P1-L3	60.0	110.0	2.500	65.9	60.0	110.0	2.500	26.4
P1-L4	206.9	110.0	2.500	91.1	600.0	110.0	1.195	68.6
P1-L5	60.0	110.0	2.500	48.5	60.0	110.0	2.500	19.4
P1-L6	101.5	110.0	2.500	8.5	101.5	110.0	2.500	3.3
P1-L7	94.8	110.0	2.500	75.2	94.8	110.0	2.500	30.1

Reaction of 4 and 2 (Case II)

Following the General Procedure using 3-chloropyridine (**4**, 815.2 mg), naphthalene (336.4 mg), (3,5-dimethylisoxazol-4-yl)boronic acid pinacol ester (**2**, 1176.5 mg). The mass for each precatalyst was: 31.1 mg **P1-L1**, 27.3 mg **P2-L1**, 29.3 mg **P1-L2**, 29.2 mg **P1-L3**, 34.0 mg **P1-L4**, 25.3 mg **P1-L5**, 22.4 mg **P1-L6**, and 21.0 mg **P1-L7**. Solution volumes were automatically sampled to achieve 0.167 M aryl halide, 0.250 M boronic acid pinacol ester, 0.333 M DBU, 0.000835-0.004175 M precatalyst-ligand, and a 5:1 THF-water ratio in the reacting droplets. The product 3,5-dimethyl-4-(pyridin-3-yl)isoxazole (**5**) was detected by UV at 270 nm. Reaction data are presented in Table S.3. Optimization results are presented in Table S.4.

Table S.3. Experimental data for reaction optimization of 4 and 2.

Experiment	Catalyst	t_{res} (s)	T (ºC)	Cat. Loading (mol%)	TON	Yield (%)
1	P1-L6	600.0	110.0	2.490	0.0	0.1
2	P1-L5	60.0	110.0	0.510	0.2	0.1
3	P1-L4	60.0	110.0	0.498	0.2	0.1
4	P1-L7	600.0	110.0	2.489	2.3	5.7
5	P1-L1	60.0	110.0	2.516	2.5	6.4
6	P1-L2	60.0	110.0	2.516	4.4	11.0
7	P1-L3	600.0	110.0	0.512	0.2	0.1
8	P2-L1	600.0	110.0	0.507	0.2	0.1
9	P1-L5	600.0	30.0	2.492	0.0	0.1
10	P1-L4	600.0	30.0	2.516	0.2	0.5
11	P1-L7	60.0	30.0	0.509	0.2	0.1
12	P1-L6	60.0	30.0	0.492	0.2	0.1
13	P1-L1	600.0	30.0	0.509	0.2	0.1
14	P1-L3	60.0	30.0	2.505	0.0	0.1
15	P2-L1	60.0	30.0	2.509	0.0	0.1
16	P1-L2	600.0	30.0	0.492	0.2	0.1
17	P2-L1	60.0	65.3	1.121	0.3	0.4
18	P1-L4	189.7	65.3	2.516	0.4	1.0
19	P1-L5	60.0	65.3	2.492	0.0	0.1
20	P1-L1	189.7	65.3	1.131	5.2	5.9
21	P1-L6	60.0	65.3	1.122	0.1	0.1
22	P1-L2	189.7	65.3	1.128	7.8	8.9
23	P1-L7	60.0	65.3	1.131	0.1	0.1
24	P1-L3	60.0	65.3	2.505	4.7	11.8
25	P1-L6	189.7	110.0	2.490	0.0	0.1
26	P1-L5	189.7	110.0	1.111	7.2	8.2
27	P1-L2	60.0	110.0	2.516	4.6	11.5
28	P1-L3	189.7	110.0	1.131	9.2	10.5
29	P2-L1	189.7	110.0	2.509	2.1	5.2
30	P1-L1	60.0	110.0	2.516	5.4	13.5
31	P1-L4	60.0	110.0	1.106	0.3	0.4
32	P1-L7	189.7	110.0	2.489	4.6	11.7
33	P1-L2	339.6	110.0	2.516	6.4	16.1
34	P1-L4	351.3	110.0	2.516	1.0	2.4
35	P1-L5	370.2	110.0	2.492	11.1	28.0
36	P1-L1	340.3	110.0	2.516	6.5	16.3
37	P1-L7	600.0	33.8	2.489	0.0	0.1
38	P2-L1	600.0	33.4	2.509	0.5	1.2
39	P1-L3	600.0	30.0	2.047	0.1	0.1
40	P1-L6	600.0	30.0	2.490	0.0	0.1
41	P1-L7	149.0	110.0	2.489	11.2	28.1
42	P1-L5	156.7	110.0	2.492	6.5	16.4
43	P1-L3	162.3	110.0	2.505	6.7	16.9
44	P1-L1	172.3	107.8	2.516	7.9	19.9

Table S.3. (cont.) Experimental data for reaction optimization of 4 and 2.

Experiment	Catalyst	t_{res} (s)	T (ºC)	Cat. Loading (mol%)	TON	Yield (%)
45	P1-L2	177.6	95.1	2.516	5.2	13.0
46	P1-L1	222.1	110.0	0.792	10.9	8.5
47	P1-L2	192.5	110.0	2.169	5.4	11.6
48	P1-L7	155.6	110.0	1.923	2.8	5.4
49	P1-L5	184.7	110.0	2.011	9.1	18.6
50	P1-L3	262.1	56.1	0.754	7.0	5.5
51	P1-L3	203.4	81.9	2.505	7.2	18.0
52	P1-L2	190.0	93.8	2.516	5.6	14.2
53	P1-L5	184.5	110.0	2.492	7.2	18.1
54	P1-L1	213.2	110.0	2.516	9.8	24.6
55	P1-L7	157.7	110.0	2.489	2.7	6.8
56	P1-L1	261.0	110.0	2.516	10.3	26.0
57	P1-L5	206.5	110.0	2.492	9.7	24.5
58	P1-L7	166.9	110.0	2.489	4.3	10.8
59	P1-L3	198.4	100.7	2.505	6.9	17.3
60	P1-L2	600.0	110.0	1.938	8.3	15.9
61	P1-L3	600.0	110.0	1.993	9.4	18.5
62	P1-L1	600.0	110.0	1.781	13.3	24.0
63	P1-L5	600.0	110.0	1.951	15.5	30.6
64	P1-L5	600.0	110.0	1.951	15.3	30.2
65	P1-L1	600.0	110.0	1.385	14.7	20.2
66	P1-L3	375.4	97.2	1.940	8.8	16.9
67	P1-L3	600.0	103.3	2.505	8.9	22.3
68	P1-L1	600.0	110.0	2.516	13.0	32.6
69	P1-L5	600.0	110.0	2.011	19.7	40.1
70	P1-L5	600.0	110.0	2.492	16.7	42.0
71	P1-L1	60.0	30.0	2.516	0.0	0.1
72	P1-L1	60.0	30.0	2.516	0.2	0.4
73	P1-L5	600.0	110.0	2.492	18.9	47.6
74	P1-L1	600.0	47.1	2.516	16.6	41.9
75	P1-L5	600.0	71.0	2.492	0.0	0.1
76	P1-L5	600.0	110.0	2.492	16.7	42.0
77	P1-L2	600.0	39.8	2.516	0.4	1.1
78	P1-L3	600.0	38.1	2.505	1.8	4.6
79	P1-L1	600.0	61.0	2.516	5.6	14.2
80	P1-L3	600.0	69.4	2.505	9.7	24.4
81	P1-L1	600.0	63.0	2.516	6.3	15.7
82	P1-L1	600.0	110.0	2.516	5.6	14.0
83	P1-L5	600.0	110.0	2.492	18.1	45.4
84	P1-L5	600.0	110.0	2.492	15.2	38.4
85	P1-L3	600.0	110.0	2.505	10.0	25.0
86	P1-L5	600.0	110.0	1.711	15.1	26.2
87	P1-L1	600.0	110.0	2.516	6.8	17.2
88	P1-L5	600.0	110.0	2.492	16.3	41.1
89	P1-L1	600.0	110.0	2.516	6.8	17.1
90	P1-L1	600.0	110.0	2.516	7.0	17.7
91	P1-L5	600.0	110.0	2.492	18.0	45.2
92	P1-L5	600.0	110.0	1.771	19.4	34.8
93	P1-L1	600.0	30.0	2.488	1.0	2.5
94	P1-L1	600.0	30.0	2.488	1.3	3.2
95	P1-L5	600.0	110.0	2.492	17.5	44.0
96	P1-L5	600.0	110.0	1.921	20.3	38.9

Table S.4. Optimal yield and TON conditions for optimization of 4 and 2.

	Yield Maximum				TON Maximum			
Catalyst	t_{res} (s)	T (ºC)	Cat. Loading (mol%)	Yield (%)	t_{res} (s)	T (ºC)	Cat. Loading (mol%)	TON
P1-L1	600.0	78.5	2.500	37.4	600.0	78.5	2.500	15.0
P2-L1	600.0	96.4	2.500	6.1	600.0	96.4	2.500	2.4
P1-L2	600.0	87.6	2.500	25.2	600.0	87.6	2.500	10.1
P1-L3	600.0	85.9	2.500	29.0	600.0	85.9	2.500	11.6
P1-L4	600.0	90.9	2.500	2.7	600.0	90.9	2.500	1.1
P1-L5	600.0	110.0	2.500	39.2	600.0	110.0	2.061	17.1
P1-L6	600.0	36.4	2.500	1.2	600.0	36.4	2.500	0.5
P1-L7	600.0	110.0	2.500	20.7	600.0	110.0	2.500	8.3

Reaction of 4 and 6 (Case III)

Following the General Procedure using 3-chloropyridine (**4**, 829.4 mg), naphthalene (325.2 mg), and 2-benzofuranboronic acid (**6**, 863.5 mg) The mass for each precatalyst was: 31.1 mg **P1-L1**, 28.9 mg **P2-L1**, 28.7 mg **P1-L2**, 30.3 mg **P1-L3**, 35.1 mg **P1-L4**, 25.2 mg **P1-L5**, 22.4 mg **P1-L6**, and 20.7 mg **P1-L7**. Solution volumes were automatically sampled to achieve 0.167 M aryl halide, 0.250 M boronic acid, 0.333 M DBU, 0.000835-0.004175 M precatalyst-ligand, and a 5:1 THF-water ratio in the reacting droplets. The product 3-(benzofuran-2-yl)pyridine (**7**) was detected by UV at 340 nm. Reaction data are presented in Table S.5. Optimization results are presented in Table S.6.

Table S.5. Experimental data for reaction optimization of 4 and 6.

Experiment	Catalyst	t_{res} (s)	T (ºC)	Cat. Loading (mol%)	TON	Yield (%)
1	P1-L4	60.0	30.0	2.513	0.1	0.2
2	P1-L2	600.0	30.0	2.494	0.1	0.2
3	P1-L1	60.0	30.0	0.510	0.3	0.2
4	P1-L5	600.0	30.0	2.511	0.1	0.2
5	P1-L6	60.0	30.0	0.499	0.3	0.2
6	P1-L7	600.0	30.0	0.501	0.3	0.2
7	P1-L3	60.0	30.0	2.512	0.1	0.2
8	P2-L1	600.0	30.0	0.509	0.3	0.2
9	P2-L1	60.0	110.0	2.515	32.8	82.6
10	P1-L5	60.0	110.0	0.508	0.3	0.2
11	P1-L4	600.0	110.0	0.514	0.3	0.2
12	P1-L6	600.0	110.0	2.496	0.1	0.2
13	P1-L3	600.0	110.0	0.503	50.6	25.4
14	P1-L7	60.0	110.0	2.504	16.9	42.2
15	P1-L2	60.0	110.0	0.510	46.3	23.6

Table S.5. (cont.) Experimental data for reaction optimization of 4 and 6.

Experiment	Catalyst	t_{res} (s)	T (ºC)	Cat. Loading (mol%)	TON	Yield (%)
16	P1-L1	600.0	110.0	2.493	36.6	91.3
17	P1-L3	189.7	110.0	1.117	77.4	86.5
18	P2-L1	189.7	110.0	2.515	34.3	86.4
19	P1-L6	189.7	110.0	2.496	0.1	0.2
20	P1-L5	60.0	110.0	1.106	4.0	4.4
21	P1-L1	189.7	110.0	2.493	31.6	78.8
22	P1-L4	189.7	110.0	1.114	2.6	2.9
23	P1-L2	60.0	110.0	2.494	34.7	86.7
24	P1-L6	60.0	65.3	1.109	0.1	0.2
25	P1-L7	60.0	110.0	2.504	19.9	49.9
26	P1-L2	189.7	65.3	1.106	3.9	4.3
27	P1-L5	189.7	65.3	2.511	2.0	5.1
28	P1-L1	60.0	65.3	1.105	6.9	7.6
29	P1-L3	60.0	65.3	2.512	8.8	22.1
30	P1-L7	189.7	65.3	1.113	0.7	0.8
31	P1-L4	60.0	65.3	2.513	0.3	0.7
32	P2-L1	60.0	65.3	1.130	6.1	6.9
33	P1-L4	600.0	105.1	2.513	1.6	4.1
34	P1-L1	600.0	110.0	2.352	32.6	76.6
35	P1-L2	600.0	110.0	2.438	33.0	80.3
36	P1-L3	600.0	110.0	2.317	37.7	87.4
37	P2-L1	600.0	110.0	2.345	34.3	80.3
38	P1-L7	600.0	110.0	2.393	20.9	49.9
39	P1-L5	600.0	71.7	1.196	0.1	0.2
40	P1-L5	600.0	48.3	0.748	0.2	0.2
41	P1-L7	124.3	110.0	1.113	9.8	10.9
42	P1-L2	202.9	110.0	1.219	51.4	62.6
43	P2-L1	160.9	110.0	1.074	55.6	59.7
44	P1-L1	123.2	110.0	1.105	77.6	85.8
45	P1-L3	600.0	83.8	1.061	48.6	51.7
46	P2-L1	60.0	110.0	1.413	31.1	43.9
47	P1-L7	60.0	110.0	1.419	24.8	35.1
48	P1-L2	60.0	110.0	1.672	53.1	88.8
49	P1-L3	60.0	110.0	1.312	59.8	78.4
50	P1-L1	60.0	110.0	1.417	61.8	87.5
51	P1-L2	186.8	110.0	2.494	30.3	75.4
52	P1-L1	149.9	110.0	2.493	32.0	79.8
53	P2-L1	186.8	103.6	2.515	33.2	83.4
54	P1-L3	179.9	110.0	2.512	34.4	86.5
55	P1-L1	60.0	110.0	1.502	56.6	85.0
56	P1-L2	60.0	110.0	1.927	43.6	84.2
57	P1-L3	137.9	110.0	1.312	60.6	79.4
58	P1-L3	60.0	110.0	1.647	49.5	81.4
59	P1-L1	177.6	110.0	1.048	77.4	81.1
60	P1-L2	188.8	110.0	1.446	49.5	71.5
61	P1-L3	211.9	110.0	1.368	59.9	82.0
62	P1-L2	239.7	110.0	1.332	51.2	68.2
63	P1-L1	253.3	110.0	1.048	83.0	87.0
64	P1-L1	108.9	110.0	1.275	65.3	83.3
65	P1-L3	92.2	110.0	1.787	50.5	90.2
66	P1-L2	66.6	110.0	2.409	35.8	86.4
67	P1-L3	192.1	110.0	1.731	48.1	83.3

Table S.5. (cont.) Experimental data for reaction optimization of 4 and 6.

Experiment	Catalyst	t_{res} (s)	T (ºC)	Cat. Loading (mol%)	TON	Yield (%)
68	P1-L1	221.3	110.0	1.077	75.9	81.7
69	P1-L2	192.7	110.0	1.871	41.5	77.7
70	P1-L1	180.1	110.0	1.303	65.1	84.9
71	P1-L2	144.0	110.0	1.899	44.5	84.6
72	P1-L3	146.6	110.0	2.038	43.0	87.7
73	P1-L1	60.0	110.0	2.493	34.4	85.9
74	P1-L3	101.5	110.0	1.926	46.9	90.4
75	P1-L2	60.0	110.0	2.126	38.3	81.4
76	P1-L1	218.5	110.0	1.105	80.8	89.4
77	P1-L2	135.7	110.0	1.814	40.5	73.5
78	P1-L1	600.0	110.0	0.935	85.6	79.9
79	P1-L3	147.9	110.0	1.591	52.1	82.9
80	P1-L2	162.9	110.0	1.701	40.0	68.0
81	P1-L3	162.1	110.0	1.675	53.8	90.1
82	P1-L1	600.0	110.0	1.218	74.3	90.6
83	P1-L3	600.0	110.0	1.424	49.3	70.1
84	P1-L2	600.0	110.0	1.417	66.4	94.1
85	P1-L3	60.0	110.0	1.926	45.4	87.6
86	P1-L1	600	110	1.090	80.1	87.3
87	P1-L2	60.0	110.0	1.757	44.3	77.9
88	P1-L3	600.0	110.0	1.787	45.3	81.0
89	P1-L2	600.0	110.0	1.616	52.3	84.5
90	P1-L1	600.0	110.0	1.303	64.4	84.0
91	P1-L1	600.0	110.0	1.218	65.4	79.6
92	P1-L2	600.0	110.0	1.587	47.2	75.0
93	P1-L3	600.0	110.0	1.787	43.6	78.0
94	P1-L3	60.0	40.2	0.503	0.3	0.2
95	P1-L3	60.0	40.1	0.503	0.3	0.2
96	P1-L3	229.2	110.0	1.619	49.6	80.1

Table S.6. Optimal yield and TON conditions for optimization of 4 and 6.

	Yield Maximum				TON Maximum			
Catalyst	t_{res} (s)	T (ºC)	Cat. Loading (mol%)	Yield (%)	t_{res} (s)	T (ºC)	Cat. Loading (mol%)	TON
P1-L1	198.1	110.0	1.697	97.6	233.2	110.0	1.170	75.1
P2-L1	186.2	110.0	1.909	78.1	186.2	110.0	1.909	40.9
P1-L2	60.0	110.0	1.853	81.6	60.0	110.0	1.853	44.0
P1-L3	198.1	110.0	1.697	87.7	198.1	110.0	1.697	51.7
P1-L4	600.0	89.1	2.330	3.5	600.0	89.1	2.330	1.5
P1-L5	600.0	86.7	2.383	5.8	600.0	86.7	2.383	2.4
P1-L6	600.0	30.0	2.500	28.7	600.0	30.0	2.500	11.5
P1-L7	165.6	110.0	2.009	40.3	165.6	110.0	2.009	20.1

Reaction of 8 and 9 (Case IV)

Following the General Procedure using 2-chloropyridine (**8**, 816.4 mg), naphthalene (327.1 mg), *N*-boc-2-pyrroleboronic acid (**9**, 1176.5 mg). The mass for each precatalyst was: 29.6 mg **P1-L1**, 27.9 mg **P2-L1**, 28.7 mg **P1-L2**, 29.4 mg **P1-L3**, 34.0 mg **P1-L4**, 25.1 mg **P1-L5**, 23.2 mg **P1-L6**, and 20.1 mg **P1-L7**. Solution volumes were automatically sampled to achieve 0.167 M aryl halide, 0.250 M boronic acid, 0.333 M DBU, 0.000835-0.004175 M precatalyst-ligand, and a 5:1 THF-water ratio in the reacting droplets. The product *tert*-butyl-2-(pyridin-2-yl)-1*H*-pyrrole-1-carboxylate (**10**) was detected by UV at 340 nm. Reaction data are presented in Table S.7. Optimization results are presented in Table S.8. Screening results at 80 °C, 97 °C, and 110 °C are presented in Table S.9.

Table S.7. Experimental data for reaction optimization of 8 and 9.

Experiment	Catalyst	t_{res} (s)	T (ºC)	Cat. Loading (mol%)	TON	Yield (%)
1	P1-L6	600.0	110.0	2.504	11.7	29.4
2	P1-L5	600.0	110.0	2.499	21.4	53.6
3	P1-L7	60.0	110.0	2.491	9.5	23.7
4	P1-L3	60.0	110.0	0.502	129.2	64.8
5	P2-L1	600.0	110.0	0.506	99.3	50.3
6	P1-L2	600.0	110.0	0.511	107.8	55.1
7	P1-L4	60.0	110.0	0.489	32.4	15.8
8	P1-L1	60.0	110.0	2.501	37.6	94.1
9	P1-L1	600.0	30.0	0.500	2.9	1.5
10	P1-L6	60.0	30.0	0.501	0.2	0.1
11	P2-L1	60.0	30.0	2.508	1.6	4.0
12	P1-L7	600.0	30.0	0.503	0.2	0.1
13	P1-L5	60.0	30.0	0.500	0.2	0.1
14	P1-L4	600.0	30.0	2.492	4.6	11.5
15	P1-L2	60.0	30.0	2.507	0.0	0.1
16	P1-L3	600.0	30.0	2.510	2.7	6.9
17	P2-L1	600.0	65.3	2.508	38.1	95.5
18	P1-L1	600.0	65.3	1.120	65.3	73.1
19	P1-L6	189.7	65.3	1.127	1.3	1.4
20	P1-L4	600.0	65.3	2.492	29.7	74.1
21	P1-L2	189.7	65.3	2.507	36.4	91.3
22	P1-L7	600.0	65.3	1.126	0.7	0.8
23	P1-L3	189.7	65.3	1.123	60.8	68.3
24	P1-L5	189.7	65.3	1.131	1.3	1.5
25	P1-L1	189.7	110.0	2.501	34.9	87.2
26	P1-L4	189.7	110.0	1.124	50.6	56.9
27	P1-L6	600.0	110.0	2.504	16.1	40.4
28	P1-L3	600.0	110.0	2.510	32.8	82.2
29	P1-L7	189.7	110.0	2.491	11.6	28.8
30	P1-L2	600.0	110.0	1.120	72.2	80.9
31	P1-L5	600.0	110.0	2.499	25.7	64.3
32	P2-L1	189.7	110.0	1.109	80.6	89.4

Table S.7. (cont.) Experimental data for reaction optimization of 8 and 9.

Experiment	Catalyst	t_{res} (s)	T (ºC)	Cat. Loading (mol%)	TON	Yield (%)
33	P1-L5	114.7	110.0	1.079	31.4	33.9
34	P1-L6	114.7	110.0	1.077	21.4	23.0
35	P1-L7	250.1	110.0	0.982	26.8	26.3
36	P2-L1	150.3	71.6	1.037	27.7	28.7
37	P1-L2	145.8	81.2	1.071	74.9	80.2
38	P1-L4	226.1	69.5	1.148	8.1	9.2
39	P1-L3	326.4	109.2	0.932	96.1	89.6
40	P1-L1	168.9	89.8	0.858	100.7	86.4
41	P1-L1	600.0	61.9	2.501	37.8	94.4
42	P1-L2	227.6	101.6	2.507	33.6	84.1
43	P1-L4	600.0	83.7	2.492	35.9	89.4
44	P1-L5	60.0	108.5	2.499	19.4	48.4
45	P1-L3	600.0	75.7	2.510	36.1	90.5
46	P2-L1	182.6	104.0	2.508	34.1	85.5
47	P1-L2	600.0	61.5	1.387	59.5	82.6
48	P1-L1	600.0	106.8	1.215	72.5	88.1
49	P1-L3	600.0	84.7	1.793	48.3	86.5
50	P2-L1	600.0	96.5	1.977	41.3	81.8
51	P1-L5	600.0	110.0	1.236	34.8	43.0
52	P1-L2	600.0	73.7	2.507	35.1	88.0
53	P1-L3	134.4	110.0	1.530	55.8	85.3
54	P2-L1	196.1	91.7	2.050	42.1	86.2
55	P1-L1	227.9	106.5	0.786	108.5	85.3
56	P2-L1	260.4	88.2	2.098	40.0	83.9
57	P1-L2	600.0	80.8	1.996	47.6	95.0
58	P1-L3	169.1	98.5	2.056	39.4	81.0
59	P1-L1	236.9	105.3	0.739	106.8	78.9
60	P1-L1	60.0	70.4	1.715	39.1	67.0
61	P1-L3	60.0	82.1	2.295	40.0	91.8
62	P1-L2	60.0	85.6	2.507	34.5	86.6
63	P1-L1	600.0	97.7	0.715	115.4	82.5
64	P1-L2	600.0	72.8	1.314	66.2	87.0
65	P1-L3	319.6	94.9	1.171	71.4	83.6
66	P1-L1	299.9	73.5	2.501	39.9	99.8
67	P1-L3	237.1	95.6	1.554	51.4	79.9
68	P1-L2	600.0	87.0	1.801	46.9	84.6
69	P1-L2	196.0	110.0	1.412	59.9	84.5
70	P1-L1	202.8	108.8	1.358	49.5	67.3
71	P1-L2	205.1	76.3	1.655	50.0	82.7
72	P1-L1	222.9	74.7	1.572	51.9	81.6
73	P1-L1	167.6	73.2	2.287	38.4	87.9
74	P1-L2	200.4	75.8	1.947	50.7	98.7
75	P1-L2	600.0	84.2	2.507	35.5	88.9
76	P1-L3	600.0	98.2	2.510	33.0	82.8
77	P1-L1	600.0	97.7	2.501	33.5	83.8
78	P1-L2	600.0	70.7	1.144	69.3	79.3
79	P1-L3	149.9	93.0	2.510	33.6	84.2
80	P1-L1	600.0	96.7	0.739	105.3	77.8
81	P1-L3	216.2	105.7	1.386	61.7	85.6
82	P1-L1	600.0	97.3	0.715	118.5	84.7
83	P1-L1	600.0	88.6	0.977	87.6	85.6
84	P1-L2	600.0	77.4	1.193	66.2	78.9

Table S.7. (cont.) Experimental data for reaction optimization of 8 and 9.

Experiment	Catalyst	t_{res} (s)	T (ºC)	Cat. Loading (mol%)	TON	Yield (%)
85	P1-L2	600.0	77.2	1.314	67.3	88.4
86	P1-L3	126.4	97.6	2.510	28.9	72.6
87	P1-L2	276.4	69.3	2.507	39.7	99.5
88	P1-L1	60.0	110.0	0.643	129.4	83.2
89	P1-L1	60.0	110.0	0.667	105.1	70.1
90	P1-L2	258.2	71.6	2.507	33.7	84.5
91	P1-L2	211.7	104.7	1.193	65.2	77.8
92	P1-L1	113.3	110.0	1.120	81.4	91.1
93	P1-L2	600.0	58.7	2.507	34.4	86.3
94	P1-L1	60.0	110.0	1.096	86.3	94.5
95	P1-L1	154.1	110.0	1.096	80.9	88.6
96	P1-L2	600.0	72.4	2.507	33.7	84.4
97	P1-L1	60.0	110.0	0.977	55.5	54.2

Table S.8. Optimal yield and TON conditions for optimization of 8 and 9.

	Yield Maximum				TON Maximum			
Catalyst	t_{res} (s)	T (ºC)	Cat. Loading (mol%)	Yield (%)	t_{res} (s)	T (ºC)	Cat. Loading (mol%)	TON
P1-L1	212.5	85.1	2.401	99.7	282.5	97.2	1.012	88.7
P2-L1	366.8	88.2	2.088	88.1	366.8	88.2	2.088	42.2
P1-L2	307.5	78.0	2.500	95.8	373.9	85.9	1.381	65.0
P1-L3	235.8	92.1	1.983	90.4	256.0	95.5	1.442	61.7
P1-L4	600.0	79.6	2.500	72.6	600.0	79.6	2.500	29.0
P1-L5	91.6	110.0	1.707	54.1	91.6	110.0	1.707	31.7
P1-L6	100.9	110.0	1.790	33.7	100.9	110.0	1.790	18.8
P1-L7	143.4	110.0	1.760	27.4	143.4	110.0	1.760	15.6

Table S.9. Experimental data for screening of 8 and 9.

Experiment	Catalyst	t_{res} (s)	T (ºC)	Cat. Loading (mol%)	TON	Yield (%)
1	P1-L1	150.0	80.0	1.018	61.5	62.6
2	P1-L1	600.0	80.0	1.018	81.7	83.2
3	P1-L1	282.0	80.0	1.018	74.8	76.2
4	P1-L1	600.0	80.0	1.018	83.5	85.0
5	P1-L1	282.0	97.0	1.018	86.7	88.3
6	P1-L1	282.0	97.0	1.018	89.0	90.6
7	P1-L1	450.0	97.0	1.018	86.3	87.8
8	P1-L1	600.0	97.0	1.018	83.7	85.2
9	P1-L1	600.0	110.0	1.018	82.9	84.4
10	P1-L1	282.0	110.0	1.018	85.0	86.5
11	P1-L1	150.0	110.0	1.018	87.9	89.5
12	P1-L1	450.0	110.0	1.018	82.0	83.5
13	P1-L1	450.0	80.0	1.018	78.0	79.4
14	P1-L1	450.0	80.0	1.018	77.5	78.9
15	P1-L1	282.0	80.0	1.018	71.6	72.9
16	P1-L1	150.0	80.0	1.018	60.6	61.7
17	P1-L1	600.0	97.0	1.018	85.3	86.8
18	P1-L1	450.0	97.0	1.018	92.8	94.4
19	P1-L1	150.0	97.0	1.018	84.9	86.4
20	P1-L1	150.0	97.0	1.018	87.5	89.0
21	P1-L1	282.0	110.0	1.018	85.4	86.9
22	P1-L1	150.0	110.0	1.018	86.0	87.5
23	P1-L1	600.0	110.0	1.018	78.6	80.0
24	P1-L1	450.0	110.0	1.018	84.5	86.0

Optimization of Ligand Equivalents

Following the general procedure, stock solutions were prepared using **4** (816 mg), naphthalene (327 mg), and **11** (727 mg). The mass for each precatalyst was: 29.5 mg **P1-L1**, 23.7 mg **P1-L5**, and 20.4 mg **P1-L7**. Separate solutions of ligands were prepared by charging the solid (0.10 mmol) to a tapered 2 mL vial, and then adding THF (2 mL) or water (2 mL). The mass for each ligand was: 49.6 mg **L1** (in THF), 39.1 mg **L5**-HBF_4 (in THF), and 29.2 mg **L7**-HBF_4 (in water). Solution volumes were automatically sampled to achieve 0.167 M aryl halide, 0.250 M boronic acid, 0.333 M DBU, 0.0023 M precatalyst-ligand, 0.0000-0.0046 M added equivalents ligand, and a 5:1 THF-water ratio in the reacting droplets. The product 3,5-dimethyl-4-(pyridin-3-yl)isoxazole (**5**) was detected by UV at 270 nm. Reaction data are presented in Table S.10. Optimization results are presented in Table S.11.

Table S.10. Experimental data for reaction optimization of 4 and 11.

Experiment	Catalyst	*T* (ºC)	Excess Ligand Equiv.	TON	Yield (%)
1	P1-L5	110.0	2.0	3.2	13.6
2	P1-L1	110.0	2.0	5.8	24.5
3	P1-L5	110.0	0.0	21.4	30.1
4	P1-L7	110.0	2.1	1.7	6.8
5	P1-L1	110.0	0.0	14.8	20.7
6	P1-L7	110.0	0.0	8.5	11.9
7	P1-L5	30.0	2.0	0.0	0.1
8	P1-L1	30.0	2.0	2.4	9.9
9	P1-L7	30.0	2.1	0.0	0.1
10	P1-L5	30.0	0.0	0.1	0.1
11	P1-L7	30.0	0.0	0.1	0.1
12	P1-L1	30.0	0.0	7.3	10.1
13	P1-L7	65.3	0.8	0.1	0.1
14	P1-L1	65.3	0.8	8.1	19.9
15	P1-L5	65.3	0.0	0.1	0.1
16	P1-L1	65.3	0.0	11.9	16.5
17	P1-L7	65.3	0.0	0.1	0.1
18	P1-L5	65.3	0.8	0.1	0.1
19	P1-L5	110.0	0.8	18.5	45.5
20	P1-L5	110.0	0.0	24.3	34.1
21	P1-L1	110.0	0.8	12.6	30.7
22	P1-L7	110.0	0.8	7.1	17.2
23	P1-L1	110.0	0.0	18.3	25.6
24	P1-L7	110.0	0.0	8.2	11.5
25	P1-L5	110.0	0.6	17.6	39.2
26	P1-L1	110.0	0.7	9.6	22.8
27	P1-L5	110.0	0.5	20.1	41.5
28	P1-L5	110.0	0.5	18.2	37.4
29	P1-L5	110.0	0.4	19.3	38.3
30	P1-L5	110.0	0.2	20.7	35.7
31	P1-L5	110.0	0.0	27.8	39.1
32	P1-L5	110.0	0.0	30.3	42.5
33	P1-L5	110.0	0.0	31.9	44.8
34	P1-L5	110.0	0.0	34.7	48.8
35	P1-L5	110.0	0.0	29.1	40.9
36	P1-L5	110.0	0.0	33.6	47.2
37	P1-L5	110.0	0.0	31.2	43.8
38	P1-L5	110.0	0.0	31.4	44.2
39	P1-L5	110.0	0.0	27.3	38.4
40	P1-L5	110.0	0.0	27.6	38.8
41	P1-L5	110.0	0.0	26.1	36.6

Table S.11. Optimal yield and TON conditions for optimization of 4 and 11.

	Yield Maximum			TON Maximum (Per Ligand Equiv)		
Catalyst	T (ºC)	Excess Ligand Equivalents	Yield (%)	T (ºC)	Excess Ligand Equivalents	TON
P1-L1	110.0	0.276	26.5	110.0	0.276	15.1
P1-L5	110.0	0.225	42.2	110.0	0.000	29.0
P1-L7	110.0	0.328	13.4	110.0	0.328	7.3

Time-Course Evolution of 6

Following the general procedure, stock solutions were prepared using naphthalene (329 mg, no aryl halide), **6** (848 mg), and benzofuran-2-boronic acid pinacol ester (**12**, 1216 mg). Solution volumes were automatically sampled to achieve 0.250 M **6** or **12**, 0.333 M DBU, and a 5:1 THF-water ratio. The droplets were charged to the flow reactor at 30 ºC and at 110 ºC. The boronic acid **6** was detected by UV at 300 nm. The pinacol ester **12** hydrolyzed rapidly to **6** under HPLC conditions. At 110 ºC, we observed **6** to undergo rapid protodeboronation, resulting in 90% loss of the boronic acid in 10 min (Figure S.6, blue data points). Exposure of the pinacol ester **12** to the same conditions resulted in Figure S.6, red data points, indicating the combined concentration of **6** and **12** was maintained above 0.10 M throughout the 10 min.

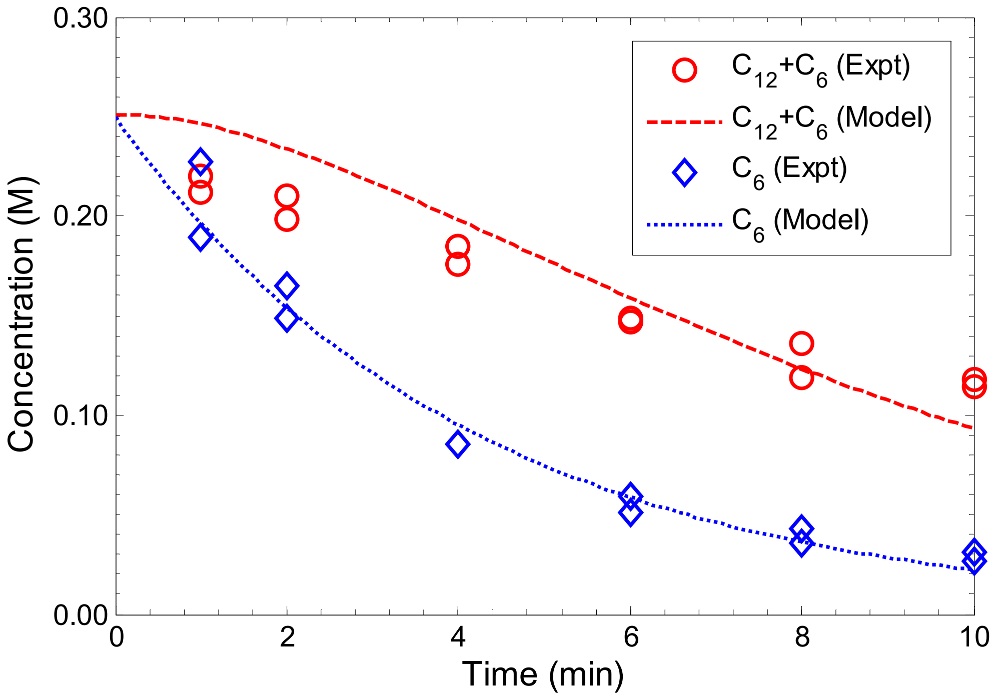

Figure S.6. Observed boronic acid concentration by HPLC. Blue curve – kinetic profile starting with 0.25 M benzofuran-2-boronic acid (6). Diamonds - droplet flow experiments. Line - best-fit model. Red curve – kinetic profile starting with 0.25 M benzofuran-2-boronic acid pinacol ester (12) yielding combined 6 and 12 observed by HPLC. Circles - droplet flow experiments. Line - best-fit model.

The conversion of boronic ester to boronic acid and from boronic acid to degradation products were each assumed to behave as first order in boronate species, given the excess of water available for hydrolysis. The kinetic equations were written as:

$$\frac{dC_{12}}{dt} = -k_1 C_{12} \tag{18}$$

$$\frac{dC_6}{dt} = k_1 C_{12} - k_2 C_6 \tag{19}$$

Equations 18 and 19 were solved to produce expressions for C_{12} and C_6 as functions of k_1 and k_2:

$$C_{12} = \left[C_{12}\right]_0 e^{-k_1 t} \tag{20}$$

$$C_6 = \left[C_6\right]_0 e^{-k_2 t} + \left[C_{12}\right]_0 \left(\frac{k_1}{k_1 - k_2}\right)\left(e^{-k_2 t} - e^{-k_1 t}\right) \tag{21}$$

$$C_6 + C_{12} = \left[C_6\right]_0 e^{-k_2 t} + \left[C_{12}\right]_0 \left(\frac{k_1 e^{-k_2 t} - k_2 e^{-k_1 t}}{k_1 - k_2}\right) \tag{22}$$

Best-fit estimates for the rate constants k_1 and k_2 were found by least-squares regression in MATLAB of the predicted C_6 and [$C_6 + C_{12}$] in comparison to the observed kinetic profiles for degradation of **6** and **12**, respectively. k_1 was estimated as 0.190 min^{-1} and k_2 was estimated as 0.241 min^{-1}, generating the model predictions in Figure S.6. These model predictions show good agreement with the kinetic profile of **6** at all time point and for the evolution of **12** at time points greater than 4 min.

The estimated rate laws and rate constants were subsequently applied to predict the concentration profiles for C_6 and C_{12} at 110 ºC, given an initial feed of 0.25 M boronic pinacol ester **12**. These profiles are shown in Figure S.7. Note that in spite of rapid hydrolysis of the pinacol ester, the concentration of the boronic acid **6** remains nearly constant in the range of 2-10 min.

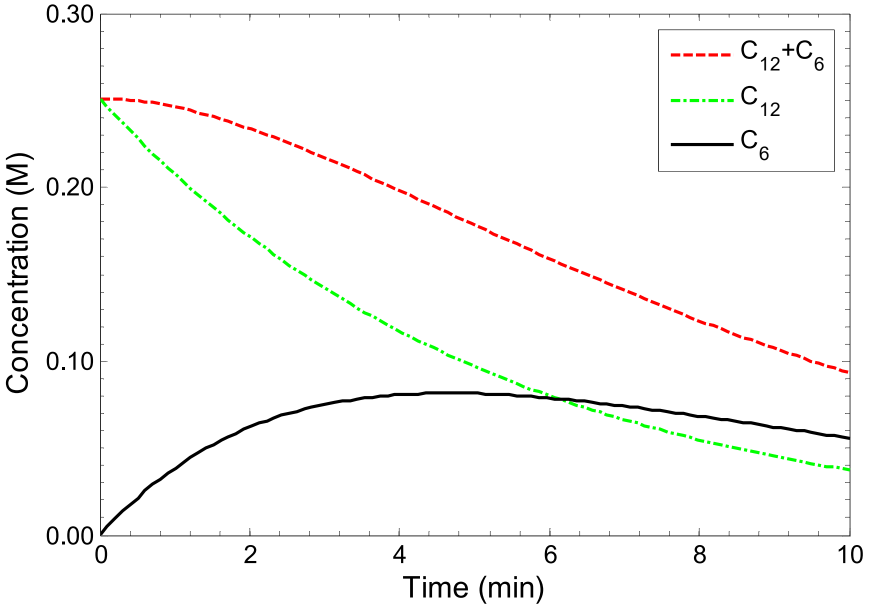

Figure S.7. Predicted boronic acid and pinacol ester concentrations starting from a feed of 0.25 M benzofuran-2-boronic acid pinacol ester (12) at 110 °C. Green curve – predicted kinetic profile of 12. Black curve – predicted kinetic profile of benzofuran-2-boronic acid (6). Red curve – predicted total concentration of 6 and 12.

Reaction of 4 and 12

Following the general procedure, stock solutions were prepared using **4** (809 mg), naphthalene (347 mg), **6** (848 mg), and benzofuran-2-boronic acid pinacol ester (**12**, 1216 mg). In a separate 5 mL volumetric flask, naphthalene (329 mg, 2.6 mmol) was diluted with THF and transferred to a 7 mL vial (this was to ensure that all droplets contained comparable amounts of internal standard in the absence of aryl halide). The mass for each precatalyst was: 30.4 mg **P1-L1** and 25.4 mg **P1-L5**. Solution volumes were automatically sampled to achieve 0.167 M aryl halide, 0.250 M **12**, 0.333 M DBU, 0.0020 M precatalyst-ligand, and a 5:1 THF-water ratio. The product **7** was detected by UV at 340 nm. The yield at 110ºC, 10 min reaction time with **P1-L1** was 88%. The yield at 110ºC, 10 min reaction time with **P1-L5** was 86%.

Product Isolation

General Procedure for the Synthesis of Authentic Samples of Biaryls

A screw-top reaction tube (16 mm × 125 mm, Fisherbrand 14-959-35A) was charged with XPhos-G3 precatalyst and capped with screw cap equipped with a Teflon/silicone septum (Thermo/National B7995-15). The tube was evacuated and flushed with argon (3 ×), and deoxygenated THF (4 mL) and deoxygenated water (1 mL) were added by syringe. Aryl halide (1.0 mmol) and 1,8-diazabicycloundec-7-ene (0.30 mL, 2.0 mmol) were added sequentially by syringe. Under argon flow, the cap was switched with a new one equipped with an unpunctured septum, and the reaction mixture was placed in an oil bath, preheated to 90 °C for 1 min. A solution of boronic acid or ester (1.5 mmol) in tetrahydrofuran (1 mL) was added by syringe (22 G needle), and heating was continued for the indicated reaction time. Subsequently, the reaction mixture was allowed to cool to ambient temperature, and Na_2SO_4 (ca. 1 g) was added to dry the reaction mixture. Solid material was removed by filtration, using EtOAc as an eluent, and the reaction mixture was concentrated. The crude product was purified by flash column chromatography using the indicated solvent system.

3,5-Dimethyl-4-(quinolin-3-yl)isoxazole (3)

The title compound was prepared following the General Procedure using 3-bromoquinoline (**1**, 208 mg), (3,5-dimethylisoxazol-4-yl)boronic acid pinacol ester (**2**, 335 mg), and 2.0% **P1-L1** (17 mg) with a reaction time of 10 min. The product was isolated as a colorless solid (**m.p.** 90-91 °C) following column chromatography using a gradient of 20% to 40% EtOAc in hexanes as the eluent. **Yield**: 162 mg, 72%. Spectroscopic data were consistent with those previously reported in the literature.[6] **^{1}H NMR** (600 MHz, $CDCl_3$) δ 8.83 (d, J = 2.2 Hz, 1H), 8.14 (d, J = 8.4 Hz, 1H), 8.03 (d, J = 1.9 Hz, 1H), 7.90 – 7.82 (m, 1H), 7.81 – 7.70 (m, 1H), 7.66 – 7.54 (m, 1H), 2.47 (s, 3H), 2.32 (s, 3H). **^{13}C NMR** (151 MHz, $CDCl_3$) δ 166.4, 158.8, 150.8, 147.4, 135.7, 130.0, 129.5, 127.8 (2 resonances), 127.4, 123.8, 113.7, 11.8, 10.9.

3,5-Dimethyl-4-(pyridin-3-yl)isoxazole (5)

The title compound was prepared following the General Procedure using 3-chloropyridine (**4**, 114 mg), (3,5-dimethylisoxazol-4-yl)boronic acid pinacol ester (**2**, 335 mg), and 1.0% **P1-L1** (8 mg) with a reaction time of 10 min. The product was isolated as a colorless solid (**m.p.** 46-47 °C) following column chromatography using a gradient of 20% to 50% EtOAc in hexanes as the eluent. **Yield**: 41 mg, 24%. Spectroscopic data were consistent with those previously reported in the literature.[6] **^{1}H NMR** (600 MHz, $CDCl_3$) δ 8.60 (dd, *J* = 4.8, 1.5 Hz, 1H), 8.54 (d, *J* = 1.5 Hz, 1H), 7.63 – 7.55 (m, 1H), 7.43 – 7.34 (m, 1H), 2.42 (s, 3H), 2.27 (s, 3H). **^{13}C NMR** (151 MHz, $CDCl_3$) δ 166.2, 158.6, 150.0, 148.9, 136.4, 126.7, 123.8, 113.6, 11.7, 10.9.

3-(Benzofuran-2-yl)pyridine (7)

The title compound was prepared following the General Procedure using 3-chloropyridine (**4**, 114 mg), 2-benzofuranboronic acid (**6**, 243 mg), and 1.0% **P1-L1** (8 mg) with a reaction time of 10 min. The product was isolated as a pale yellow solid (**m.p.** 70-72 °C) following column chromatography using a gradient of 20% to 50% EtOAc in hexanes as the eluent. **Yield**: 35 mg, 18%. Spectroscopic data were consistent with those previously reported in the literature.[7] **^{1}H NMR** (600 MHz, $CDCl_3$) δ 9.12 (s, 1H), 8.59 (d, *J* = 3.6 Hz, 1H), 8.18 – 8.09 (m, 1H), 7.62 (d, *J* = 7.7 Hz, 1H), 7.55 (d, *J* = 8.2 Hz, 1H), 7.38 (dd, *J* = 7.9, 4.8 Hz, 1H), 7.36 – 7.30 (m, 1H), 7.27 (dd, *J* = 11.7, 3.2 Hz, 1H), 7.13 (s, 1H). **^{13}C NMR** (151 MHz, $CDCl_3$) δ 155.2, 153.1, 149.5, 146.6, 132.0, 128.9, 126.8, 125.1, 123.7, 123.4, 121.3, 111.5, 102.9.

tert-Butyl-2-(pyridin-2-yl)-1*H*-pyrrole-1-carboxylate (10)

The title compound was prepared following the General Procedure using 2-chloropyridine (**8**, 114 mg), *N*-boc-pyrrolyl-2-boronic acid (**9**, 317 mg), and 1.0% **P1-L1** (8 mg) with a reaction time of 5 min. The product was isolated as a pale yellow solid (**m.p.** 52-53 °C) following column chromatography using a gradient of 0% to 12.5% EtOAc in CH_2Cl_2 as the eluent. **Yield**: 239 mg (avg. of two runs), 98%. Spectroscopic data were consistent with those previously reported in the literature.[8] **^{1}H NMR** (400 MHz, $CDCl_3$) δ 8.61 (ddd, *J* = 4.9, 1.8, 0.9 Hz, 1H), 7.68 (td, *J* = 7.7, 1.8 Hz, 1H), 7.39 (dt, *J* = 7.9, 1.0 Hz, 1H), 7.36 (dd, *J* = 3.3, 1.7 Hz, 1H), 7.19 (ddd, *J* = 7.6, 4.9,

1.2 Hz, 1H), 6.41 (dd, *J* = 3.3, 1.7 Hz, 1H), 6.24 (t, *J* = 3.3 Hz, 1H), 1.35 (s, 9H). **^{13}C NMR** (101 MHz, $CDCl_3$) δ 153.0, 149.4, 148.9, 135.9, 134.1, 123.7 (2 resonances), 121.9, 115.8, 110.7, 83.8, 27.7.

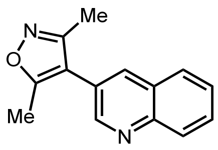
N
O
Me
Me
N

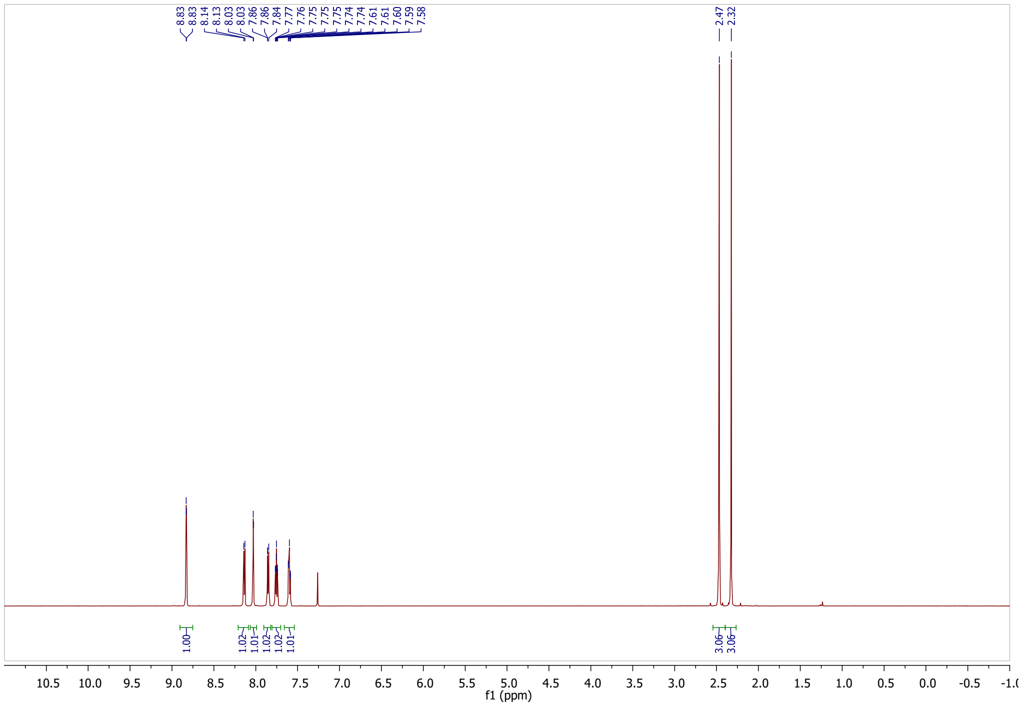
8.83
8.83
8.14
8.13
8.03
8.03
7.86
7.84
7.77
7.76
7.75
7.75
7.74
7.74
7.61
7.60
7.59
7.58
2.47
2.32
1.00
1.02
1.01
1.02
1.02
1.01
3.06
3.06
f1 (ppm)

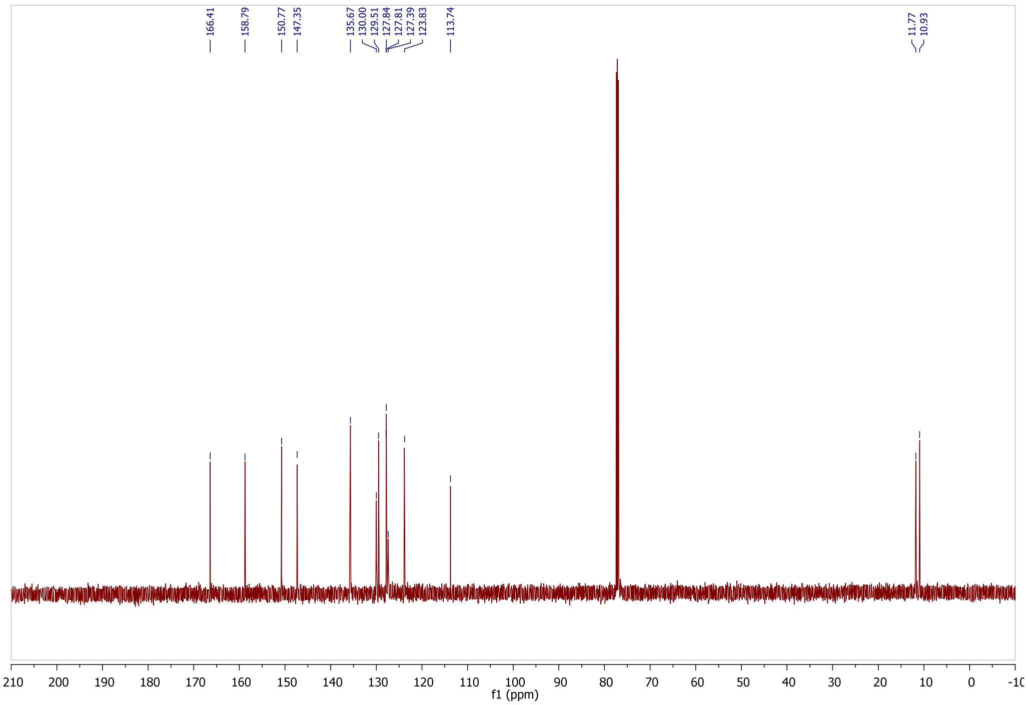
166.41
158.79
150.77
147.35
135.67
130.00
129.51
127.84
127.81
127.39
123.83
113.74
11.77
10.93
f1 (ppm)

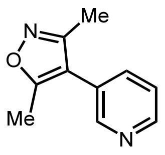
Me
N
O
Me
N

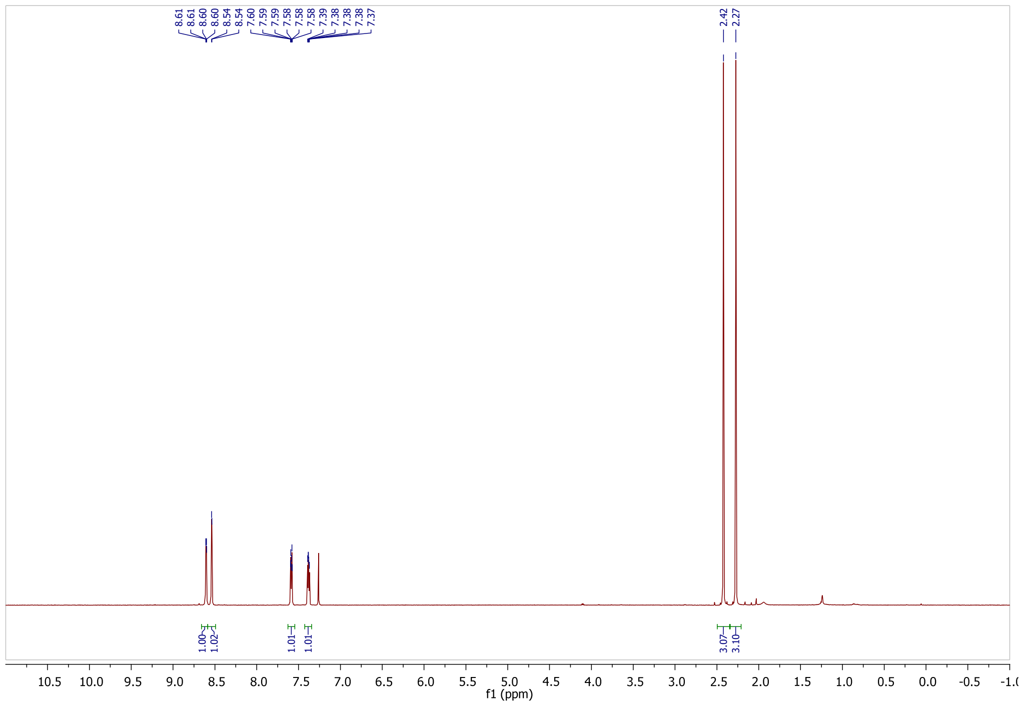
8.61
8.61
8.60
8.60
8.54
8.54
7.60
7.59
7.59
7.58
7.58
7.58
7.39
7.38
7.38
7.38
7.37
2.42
2.27
1.00
1.02
1.01
1.01
3.07
3.10
f1 (ppm)

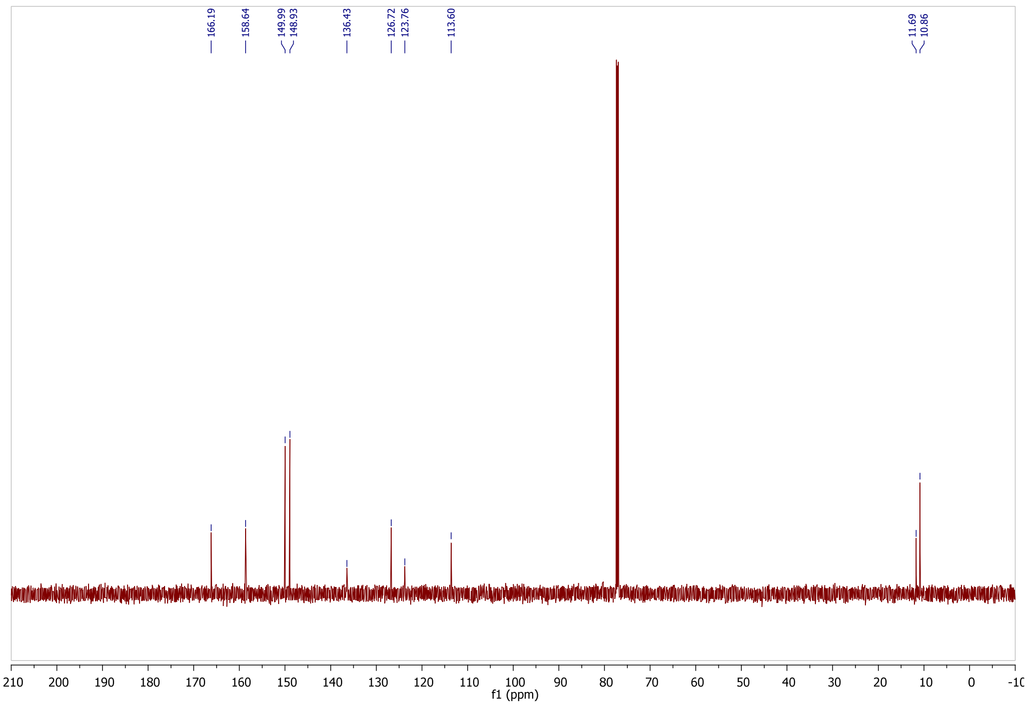
166.19
158.64
149.99
148.93
136.43
126.72
123.76
113.60
11.69
10.86
f1 (ppm)

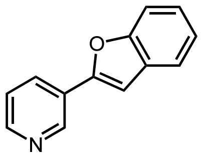

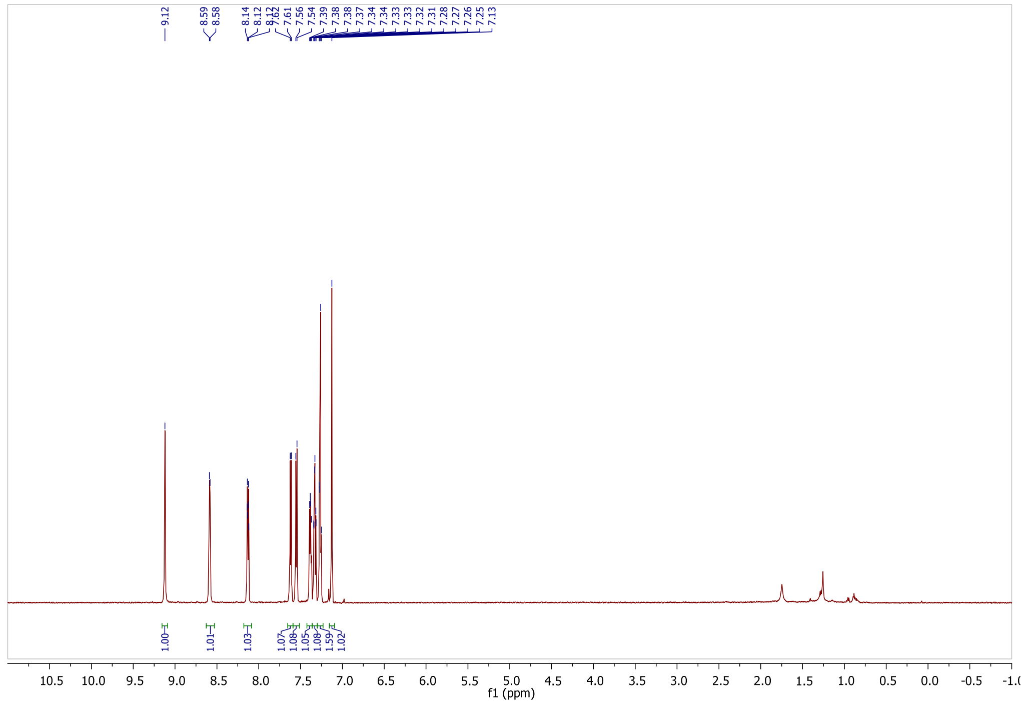

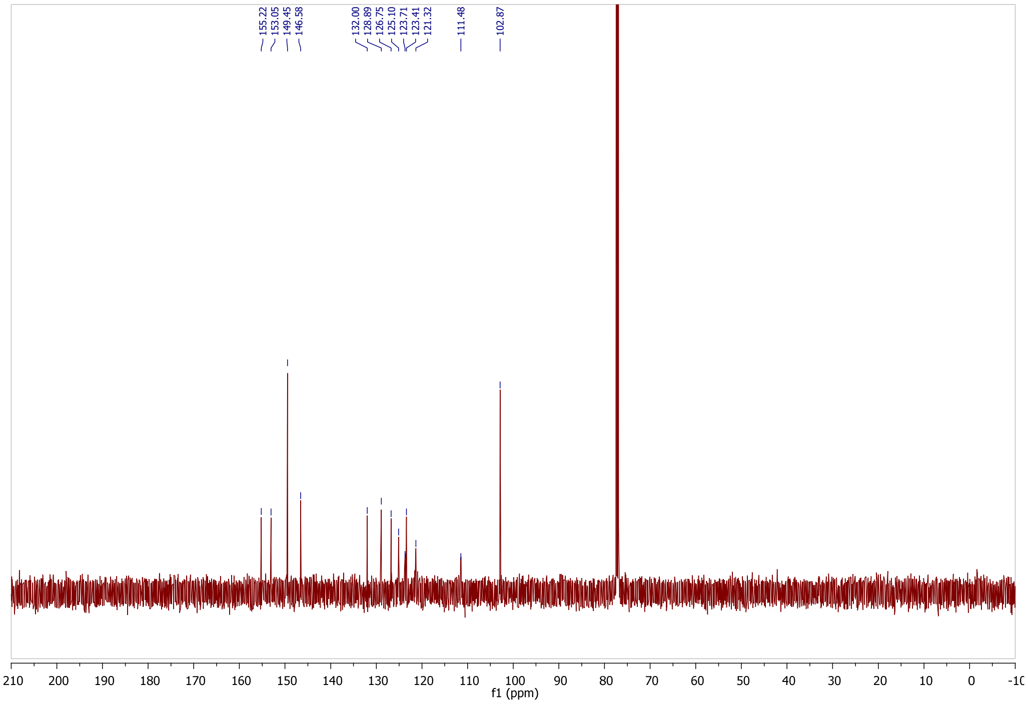

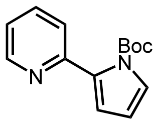
Boc
N
N

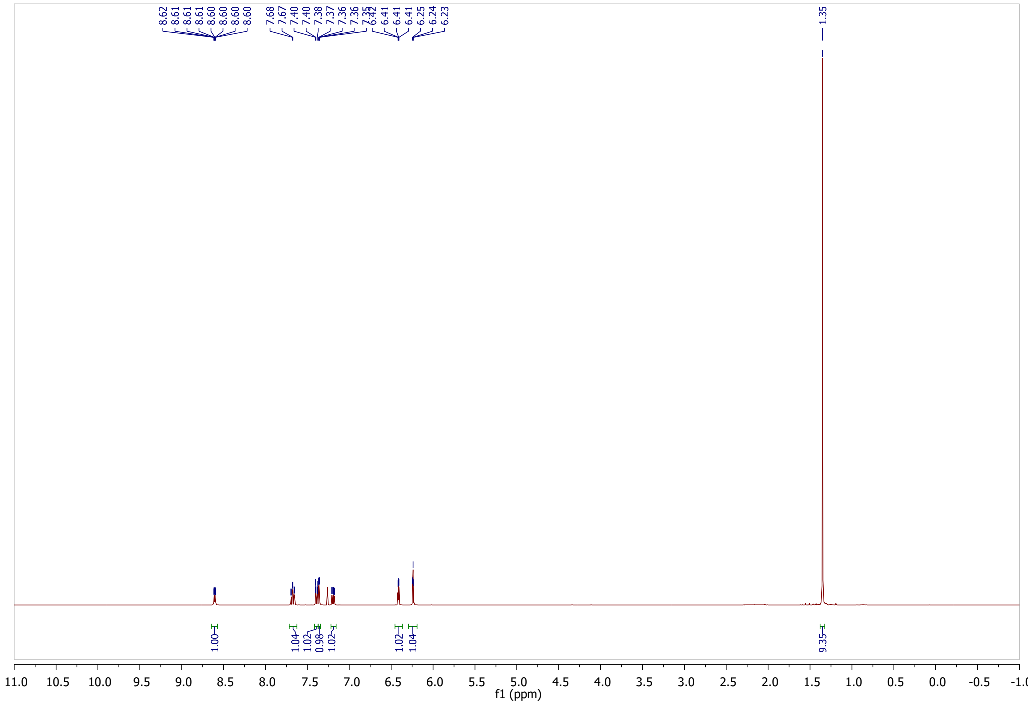
8.62
8.61
8.61
8.61
8.60
8.60
8.60
8.60
7.68
7.67
7.40
7.40
7.38
7.37
7.36
7.36
6.41
6.41
6.41
6.25
6.24
6.23
1.35
1.00
1.04
1.02
0.98
1.02
1.02
1.04
9.35
11.0
10.5
10.0
9.5
9.0
8.5
8.0
7.5
7.0
6.5
6.0
5.5
5.0
4.5
4.0
3.5
3.0
2.5
2.0
1.5
1.0
0.5
0.0
-0.5
f1 (ppm)

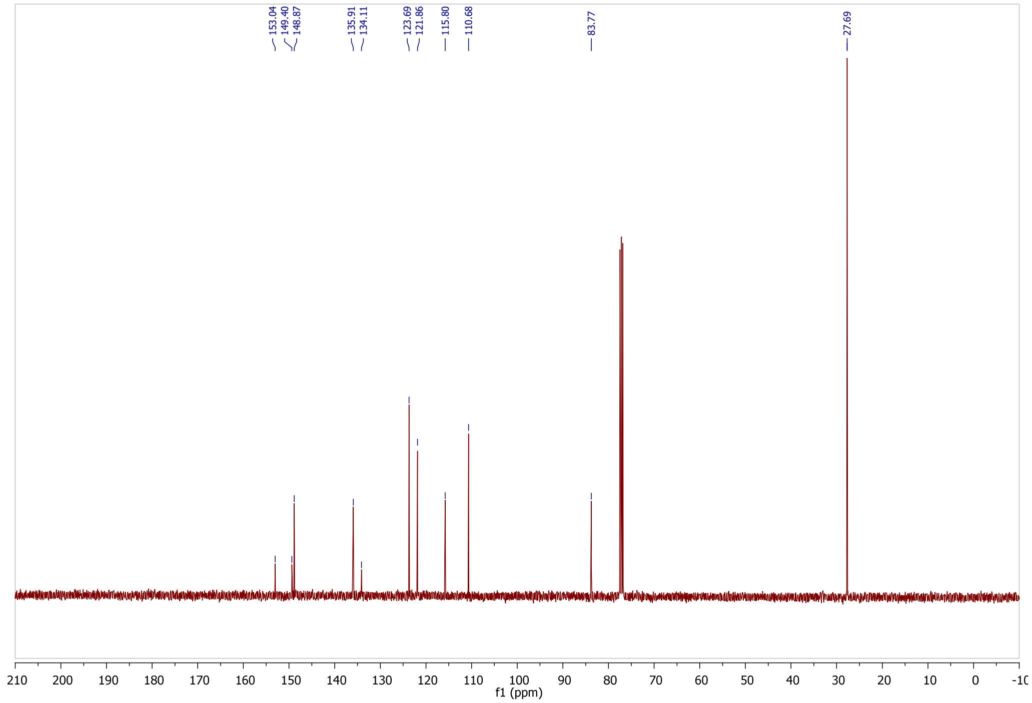
153.04
149.40
148.87
135.91
134.11
123.69
121.86
115.80
110.68
83.77
27.69
210
200
190
180
170
160
150
140
130
120
110
100
90
80
70
60
50
40
30
20
10
0
f1 (ppm)

References

(1) M. A. Burns, C. H. Mastrangelo, T. S. Sammarco, F. P. Man, J. R. Webster, B. N. Johnsons, B. Foerster, D. Jones, Y. Fields, A. R. Kaiser, D. T. Burke, *Proc. Natl. Acad. Sci. U. S. A.*, 1996, **93**, 5556.

(2) A. C. Atkinson, A. N. Donev, *Optimum Experimental Design*; Clarendon Press; Oxford University Press: Oxford, New York, 1992.

(3) J. A. Dumesic, D. F. Rudd, L. M. Aparicio, J. E. Rekoske, A. A. Trevino, *The Microkinetics of Heterogeneous Catalysis*; American Chemical Society: Washington, D.C., 1993.

(4) J. V. Beck, K. J. Arnold, *Parameter Estimation in Engineering and Science*; Wiley: New York, 1977.

(5) M. H. Quenouille, *J. Roy. Stat. Soc. B*, 1949, **11**, 68.

(6) Y. Fall, C. Reynaud, H. Doucet, M. Santelli, *Eur. J. Org. Chem.*, 2009, **24**, 4041.

(7) S. E. Denmark, R. C. Smith, W. T. T. Chang, J. M. Muhuhi, *J. Am. Chem. Soc.*, 2009, **131**, 3104.

(8) M. F. Semmelhack, A. Chlenov, D. M. Ho, *J. Am. Chem. Soc.*, 2005, **127**, 7759.
